# Binding of SU(VAR)3-9 Partially Depends on SETDB1 in the Chromosomes of *Drosophila melanogaster*

**DOI:** 10.3390/cells8091030

**Published:** 2019-09-05

**Authors:** Daniil A. Maksimov, Dmitry E. Koryakov

**Affiliations:** 1 Institute of Molecular and Cellular Biology SB RAS, 630090 Novosibirsk, Russia; 2 Epigenetics Laboratory, Department of Natural Sciences, Novosibirsk State University, 630090 Novosibirsk, Russia

**Keywords:** SU(VAR)3-9, SETDB1, salivary glands, female germline, piRNA clusters, heterochromatin, chromosome 4, *Drosophila*

## Abstract

H3K9 methylation is known to play a critical role in gene silencing. This modification is established and maintained by several enzymes, but relationships between them are not fully understood. In the present study, we decipher the interplay between two *Drosophila* H3K9-specific histone methyltransferases, SU(VAR)3-9 and SETDB1. We asked whether SETDB1 is required for targeting of SU(VAR)3-9. Using DamID-seq, we obtained SU(VAR)3-9 binding profiles for the chromosomes from larval salivary glands and germline cells from adult females, and compared profiles between the wild type and SETDB1-mutant backgrounds. Our analyses indicate that the vast majority of single copy genes in euchromatin are targeted by SU(VAR)3-9 only in the presence of SETDB1, whereas SU(VAR)3-9 binding at repeated sequences in heterochromatin is largely SETDB1-independent. Interestingly, piRNA clusters 42AB and 38C in salivary gland chromosomes bind SU(VAR)3-9 regardless of SETDB1, whereas binding to the same regions in the germline cells is SETDB1-dependent. In addition, we compared SU(VAR)3-9 profiles in female germline cells at different developmental stages (germarium cells in juvenile ovaries and mature nurse cells). It turned out that SU(VAR)3-9 binding is influenced both by the presence of SETDB1, as well as by the differentiation stage.

## 1. Introduction

Eukaryotic genomes are formed by a complex mosaic of single copy genes and repeated sequences. Some genes are active across all cell types and developmental stages, whereas other genes become activated only in a specific tissue or for a narrow period of time. Likewise, transcription of repeated DNA sequences is typically tightly controlled, and most of the time they must remain silent. Maintenance of the appropriate chromatin state, either active or inactive, occurs via epigenetic mechanisms and relies on the balance of epigenetic marks such as methylated DNA residues, as well as various post-translationally modified amino acid residues in the context of histone molecules.

DNA methylation has been found in most organisms ranging from bacteria to mammals [1]. Interestingly, whereas methylated DNA sequences represent a significant proportion of the genome in mammals, in fruit flies this is not the case, and only trace amounts of methylated DNA are detectable [2]. Yet another epigenetic system based on histone modifications presents a far greater diversity of molecular species. Methyl, acetyl, phosphate, ADP-ribosyl groups, as well as ubiquitin and SUMO molecules have been described to be attached to the lysine, arginine, serine, threonine and other amino acid residues across the histones [3]. This epigenetic system is also conserved among various organisms from single cell infusoria and fungi to worms, insects, plants, and vertebrates.

Individual histone modification is typically involved in the coordination of a specific cellular process or pathway. For instance, histone H4K16 acetylation in fruit flies has a key role in controlling hyperactivation of a single male X chromosome [4]. Similarly, in mouse embryonic stem cells, H4K16ac is a marker of active promoters and enhancers [5]. Nonetheless, the processes associated with histone H3K9 methylation are much more diverse. On the one hand, this histone mark is directly or indirectly involved in gene silencing, such as heterochromatin formation and DNA methylation in fungi, plants, and animals [6,7], programmed DNA elimination in infusoria [8], genomic imprinting in *Planococcus* [9], and DNA underreplication in *Drosophila* polytene chromosomes [10]. On the other hand, this same histone mark has also been reported to contribute to transcription elongation and alternative splicing [11,12,13].

Histone H3K9 residue is methylated by the enzymes belonging to three subfamilies: SU(VAR)3-9-like, SETDB1/ESET, and G9a/GLP. They share the substrate and the presence of a SET-domain that is responsible for the transfer of methyl groups to the substrate. Besides the catalytic domain, SU(VAR)3-9-like proteins have pre-SET and post-SET domains; G9a/GLP subfamily members have pre-SET, post-SET domains and Ankyrin repeats; SETDB1/ESET proteins have a bifurcated SET-domain [6]. Representatives of all these protein subfamilies have been identified across a broad range of organisms, yet their evolutionary trajectories have a number of distinctive features. Whereas fruit flies have a single *Su(var)3-9*-like subfamily member gene *Su(var)3-9*, and humans have two (*SUV39H1* and *SUV39H2*), *Arabidopsis* has a striking number of 10 orthologous genes [14,15,16,17]. SETDB1 orthologs have been identified in both vertebrates and invertebrates, but so far have not been found in fungi and *Arabidopsis* [6,16].

Despite sharing the same substrate specificity, the members of these three subfamilies of histone methyltransferases have distinct biological functions and chromosomal distribution. In line with this, mutations in the genes encoding these proteins have distinct phenotypes. In *Drosophila*, most of the SU(VAR)3-9 protein is localized to the pericentric heterochromatin, where it is responsible for the methylation of ~80% of H3K9me2 [17,18]. SETDB1 is largely confined to the chromosome 4, and most of the H3K9me2 in this location is SETDB1-dependent [19,20]. When germarium cells from *Drosophila* ovary were analyzed, the immunostaining signals for SU(VAR)3-9 and SETDB1 showed no overlap [21]. SETDB1 is critical for normal progression of oogenesis where it controls germline stem cell maintenance and differentiation, so the females lacking SETDB1 are sterile [22,23,24,25]. In contrast, *Su(var)3-9* mutants are viable and fertile [17,26], although a significant proportion of mutant embryos fail to reach larval stages, and not all the mutants may develop to become pupae or imagoes [27].

Despite the seemingly unrelated functions, localization and phenotypes, a number of observations suggest that SU(VAR)3-9 and SETDB1 may directly or indirectly interact. For instance, in *Drosophila* salivary gland polytene chromosomes the region 31B–E and the chromosome 4 display largely overlapping profiles of SU(VAR)3-9 and H3K9me2 binding, even though the responsible histone methyltransferase at these sites is SETDB1, not SU(VAR)3-9 [28]. Also, the sets of genes that are known to be affected by SU(VAR)3-9 or SETDB1 are very similar, both genome-wide and in the context of the chromosome 4 [29]. Finally, in mammals, a subset of methyltransferases SUV39H1 and SETDB1 have been described to form a multimeric complex [30].

In our study, we wanted to analyze the possible interaction between these proteins in more detail and asked whether SETDB1 is universally required for SU(VAR)3-9 binding to chromosomes. To address this question, we profiled SU(VAR)3-9 distribution in mutants lacking SETDB1 and matched it to the wild type situation. Further, in order to understand whether the cellular context (somatic vs. germline) may play a role, two contrasting tissues have been analyzed, i.e., larval salivary glands and ovarian germline cells from adults.

## 2. Materials and Methods

### 2.1. DNA Constructs

We used fly stocks containing four DNA constructs. Two of them encode *E. coli* DNA methyltransferase Dam under the control of either minimal or full-length *hsp70* promoter. The other two constructs encode SU(VAR)3-9-Dam chimeric protein under the same promoter elements, with SU(VAR)3-9-coding part placed upstream and in frame with the Dam-encoding sequence. The constructs driven by the minimal *hsp70* promoter were used for the analysis of salivary glands, while full-length *hsp70* promoter was used for the DamID analysis in female germline cells, because minimal *hsp70* promoter is inactive in these cells [31]. Special stop-cassettes are placed between the full-length promoter and the *Dam*/*Su(var)3-9-Dam* modules. Description of the constructs was published previously in [28].

### 2.2. Fly Stocks

In order to obtain SU(VAR)3-9 binding profiles in the absence of SETDB1, mutants in the *eggless* gene encoding SETDB1, *egg^1473^* (hereafter *egg*) were used. Viability of *egg* homo- and hemizygotes is greatly compromised, but rare escapers survive to adulthood and display a female-sterile phenotype [22,24]. The numbers of mutant larvae and adults were sufficient to collect the material for DamID.

To analyze the material from third-instar larval salivary glands, animals from the following stocks lacking the stop-cassette (Δt) in the transgene were used:

*y w* attP18[mini-*hsp70*-Δt-*Dam*]; *egg^1473^/TSTL*
*y w* attP18[mini-*hsp70*-Δt-*Su(var)3-9-Dam*]; *egg^1473^/TSTL*

These animals express Dam or SU(VAR)3-9-Dam ubiquitously, and presence of the dominantly marked *TSTL* balancer allows easy discrimination between homozygous *egg* mutant and heterozygous larvae.

For germline-focused experiments, we used the flies carrying a full-length *hsp70* promoter and a stop-cassette (t). The latter was removed by crossing to the flies expressing *nanos*-driven CRE:

*y w* attP18[full-*hsp70*-t-*Dam*]; *Df(2R)Dll-MP*/*Cy*
*y w* attP18[full-*hsp70*-t-*Su(var)3-9-Dam*]; *Df(2R)Dll-MP*/*Cy*
*y w*; *nanos*-*CRE egg^1473^*/*Cy*

In the female progeny obtained, removal of the stop-cassette occurred exclusively in the germline cells, but not in the somatic cells of the ovary. To keep the mutant background, *nanos-CRE* transgene was recombined onto the *egg^1473^* chromosome by conventional crosses, whereas *Df(2R)Dll-MP* (breakpoints 60E1-2; 60E6) overlapping the *egg* gene was introduced into the stocks with Dam/SU(VAR)3-9-Dam transgenes. Females lacking *Cy* marker at the age of 1–2 days post-eclosion were selected among the progeny of the crosses. These females were hemizygous for *egg*, and expression of the *Dam*/*Su(var) 3-9-Dam* transgenes was restricted to the germline.

In *egg* mutant females, ovaries fail to develop normally and lack egg chambers with nurse cells [21,22]. For this reason, we had to resort to comparison of SU(VAR)3-9 in *egg* mutants to that at the earliest stage of ovarian development in wild type female flies, i.e., in juvenile ovaries. Such tissue was collected from freshly eclosed (<1 hour females. The following stocks were used for this purpose:

*y w* attP18[full-*hsp70*-t-*Dam*]; +; +
*y w* attP18[full-*hsp70*-t-*Su(var)3-9-Dam*]; +; +
*y w*; *nanos-CRE*; +

All flies were kept at 18 °C on standard fly food. Organs were excised and collected in PBS. For each sample, 25 salivary glands or 30 whole ovaries were collected. For salivary glands, three biological replicates for Dam and SU(VAR)3-9-Dam samples were done. For germline, two biological replicates were done.

### 2.3. Tissue-Specific DamID-Seq Technique

We followed the tissue-specific DamID protocol described in detail in [32]. Below we outline only the concept of the procedure. Transgenic animals express either Dam, or SU(VAR)3-9-Dam fusion protein. The former protein is of bacterial origin and lacks specific binding sites in the *Drosophila* genome. The latter protein associates with the chromatin either specifically via its SU(VAR)3-9 part, or non-specifically, via Dam. In both Dam and SU(VAR)3-9-Dam, Dam part functions to methylate adenines in the GATC sites nearest to the position where binding has occurred. In *Drosophila*, cytosine or adenine methylation is only present in trace amounts in embryos, and adenine methylation takes place out of the GATC context [33,34]. Hence, whenever GATC site is methylated at an adenine, this may have only resulted from nearby binding of either Dam or SU(VAR)3-9-Dam. Thus, DNA methylation at GATC sites is used as readout of the distribution of the protein of interest. Importantly, expression of Dam-only constructs serves to provide a control for non-specific Dam binding, which reflects the local chromatin accessibility. Expression of SU(VAR)3-9-Dam fusion protein produces a composite signal of specific SU(VAR)3-9 and non-specific Dam binding.

The collected material (salivary glands or ovaries) was processed for genomic DNA isolation using standard phenol/chloroform extraction, followed by the special protocol for selective amplification of the fragments that are flanked by methylated GATC sites on both ends. The DamID libraries obtained were sequenced using Illumina MiSeq platform according to the manufacturer’s instructions. Table S1 shows strong correlations between technical replicates for each sample, with lower correlations between different biological samples. The reads were fed into the custom bioinformatic pipeline that filter the regions of specific SU(VAR)3-9 binding from nonspecific Dam binding [32]. As a result, a genome-wide distribution profile is constructed, which shows the significance values of SU(VAR)3-9 enrichment (above X axis) or Dam enrichment (below X axis) at each genomic fragment between two neighboring methylated GATC sites (−log_10_(*p*) scale). Black horizontal lines above the X axis (see appropriate figures below) denote threshold values with false discovery rate (FDR) <5%. Only the peaks exceeding the threshold value were used in all of our downstream analyses. Notably, the profiles shown have distinct threshold values, but for comparative purposes a common threshold value, —the largest among the samples compared—was used.

### 2.4. Availability of Data

DamID-seq profiles have been deposited in the GEO under accession number GSE134250.

## 3. Results

### 3.1. SU(VAR)3-9 Binding with Single Copy Genes is Mostly SETDB1-Dependent

In order to understand to which extent SETDB1 is needed for SU(VAR)3-9 binding to chromatin, we used five types of samples representing different tissues and genotypes. Specifically, we constructed SU(VAR)3-9 binding profile in the salivary glands of females that were mutant for *egg* (SG *egg*), and compared it to the distribution of SU(VAR)3-9 in wild type chromosomes (SG wt) reported earlier [28]. We also profiled SU(VAR)3-9 in the chromosome of wild type nurse cells (NC wt) [28], however, given that ovaries in *egg* females do not develop and lack nurse cells [21,22], we needed to match the SU(VAR)3-9 binding profile to the wild type situation, wherein the differentiation stage is the closest to the material collected from *egg* ovaries (OV *egg*). For this purpose, juvenile ovaries from freshly eclosed females were used (OV wt), as they are largely composed of the germarium cells.

Genome-wide, all SU(VAR)3-9 profiles appear similar across all samples and have most of the peaks in the pericentric heterochromatin, with significantly fewer peaks in the euchromatin (Figure S1). Detailed analysis of SU(VAR)3-9 targets, however, indicates that the profiles are not identical, and *egg* mutation does result in SU(VAR)3-9 re-localization. To help correctly resolve the situations with nested genes, targets analyzed here were defined as protein coding genes with at least one coding exon significantly enriched by SU(VAR)3-9 regardless of the binding status of the intronic sequences and UTRs.

**Figure 1:**
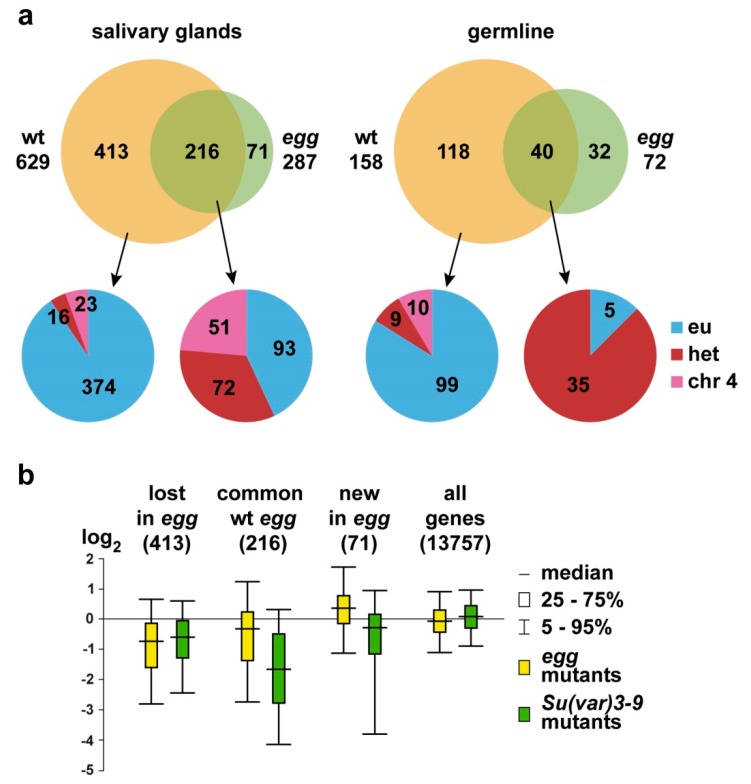
(**a**) Venn-diagrams indicating the numbers of SU(VAR)3-9 targets in the chromosomes from salivary glands and germline of wild type (wt) and *egg* mutants (upper row); distribution of SU(VAR)3-9 targets showing distinct response to *egg* mutation across euchromatin, heterochromatin, and the chromosome 4 (bottom row). (**b**) Changes in H3K9me2 binding in mutants (*egg* or *Su(var)3-9*) in comparison with wild type across the thee groups of genes, as well as genome-wide. Gene counts in each group are shown in brackets. Box-plot diagram is based on the original data on H3K9me2 distribution in the salivary gland chromosomes [35]. Chromosome-wide H3K9me2 enrichment across 30 bp intervals was calculated as log_2_(IP/input) and median values for each gene were defined. Box plots show the range of values obtained by subtracting the median gene enrichment values in wild type animals from those in mutants.

In SG wt, SU(VAR)3-9 was significantly bound to 629 genes. In *egg* mutants, the binding is reduced to 287 genes, of which 216 correspond to the gene targets in SG wt, and 71 representing novel ones. SU(VAR)3-9 binding is abrogated for the genes residing in the region 31B-E of the chromosome 2L (Figure S2). This is an interesting genomic region characterized by high density of SU(VAR)3-9 binding peaks in salivary glands, but not other cell types [28]. This region is also enriched for H3K9me2, which is laid by SETDB1 rather than by SU(VAR)3-9 [35]. In OV, of the 158 SU(VAR)-3-9-positive genes detected in wild type females, only 40 genes remain present in the *egg* mutants, and 32 more novel targets appear. Thus, roughly 2/3 and 3/4 of all the SU(VAR)3-9 gene targets are SETDB1-dependent in salivary gland and female germline chromosomes, respectively (Figure 1a; Table S2).

**Figure 2:**
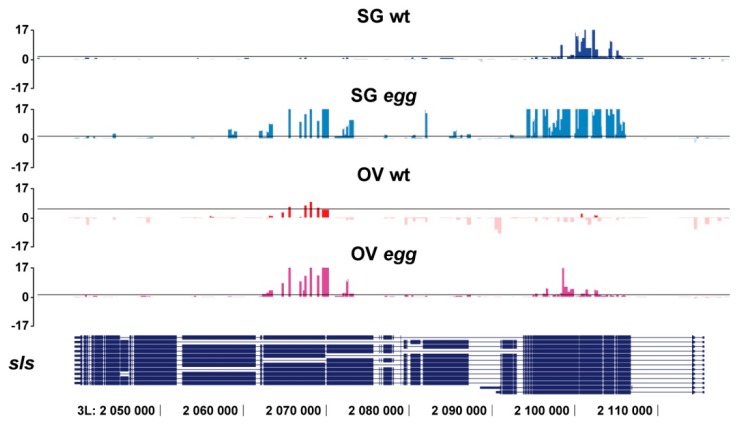
SU(VAR)3-9 profiles in the *sls* gene in salivary glands (SG) and germline of whole juvenile ovaries (OV) from wild type (wt) and *egg* mutants. Data for SG wt are taken from [28], other profiles are obtained in this study. Significance of binding of SU(VAR)3-9-Dam (values above X axis) or Dam (values below X axis) as a −log_10_ of *p*-value is plotted on the Y axis. Black horizontal lines denote threshold *p*-value corresponding to the FDR <5%. Genomic coordinates on the X axis and intron/exon structure of the *sls* gene correspond to the BDGP Release 6.

Upon closer inspection of the gene targets that formally appear SETDB1-independent, the influence of *egg* is still noticeable. In mutant background, the regions of significant SU(VAR)3-9 binding may re-distribute within the gene unit. To illustrate this, *sls* gene has two SU(VAR)3-9 binding regions: in SG wt, these include common 5′-end exons and in OV wt these include alternative exons on the 3′-end of the gene. In *egg* mutants, both regions are SU(VAR)3-9-positive in salivary glands and germline (Figure 2). More examples are found in profiles constructed for the salivary gland chromosomes. In wild type animals, *trol* gene has a single SU(VAR)3-9 peak next to the two alternative exons, whereas in *egg* mutants additional SU(VAR)3-9 signals appear in the common exons found at the 3′-end of the gene, where it is normally absent. Yet another gene, *rg* is targeted by SU(VAR)3-9 both in the wild type and mutant backgrounds, however in the latter case, numerous binding sites within the gene become detectable. In contrast, *SK* gene displays reduced density of SU(VAR)3-9 peaks in *egg* animals (Figure S3).

We analyzed H3K9 methylation status in *egg* mutants for the three groups of genes whose association with SU(VAR)3-9 is differentially affected by *egg*. H3K9me2 profiles were retrieved from the published dataset for salivary glands, a tissue displaying relatively uniform cellular composition [35]. Taking into account that ovaries are formed by complex cell populations of both somatic and germline origin, the H3K9me2 profile for the whole ovaries is not representative of the germline and therefore cannot be used in our analysis. Box-plots (yellow in each pair on Figure 1b) show the change in H3K9me2 enrichment for genes in *egg* mutants vs. wild type animals. Values below and above the X axis indicate that methylation levels are decreased/increased in the mutant background. All three sets of genes analyzed are significantly different from the genome-wide average (“all genes”) as well as from each other (*p* << 0.001; Table S3). Notably, most of the genes that lose SU(VAR)3-9 binding in *egg* animals, concomitantly display pronounced reduction in H3K9me2 levels. Essentially the same trend is observed for the SETDB1-independent gene set, albeit the magnitude of the effect is lower. For the genes that recruit SU(VAR)3-9 only in *egg* mutants (“new in *egg*” category), H3K9me2 is increased. Overall the dynamics of H3K9me2 is positively correlated with the presence of SU(VAR)3-9, although this does not apply for the SETDB1-independent gene dataset.

Next, we proceeded to analyze the changes in H3K9 methylation in the same sets of genes in *Su(var)3-9* mutants (Figure 1b, green box-plot in each pair). As above, all three groups differ significantly from the genome-wide average (*p* << 0.001; Table S3). Pairwise comparisons of box-plot data for different mutants (yellow with green box-plots) within each gene set shows that two categories (“lost in *egg*” and “all genes”) have very similar distributions, unlike the remaining two groups. Yet, despite the visual similarity, the box-plots for “all genes” differ significantly (*p* << 0.001), whereas for the “lost in *egg*” group the difference does not reach significance (*p* > 0.01; Table S3).

We then focused on the analysis of the groups “lost in *egg*” and “common wt *egg*”. To construct the box-plot diagram (Figure 1b), each gene was assigned a value obtained by subtracting the methylation enrichment level in wild type from the mutant background (*egg* or *Su(var)3-9*). By further subtracting “*Su(var)3-9* value” from “*egg* value”, we observed that for most of the “lost in *egg*” genes the results centered around zero (Figure S4). Namely, about 60% of genes are found within the −0.5/+0.5 range, with over 80% genes being distributed between −1 and +1. These data indicate that in the “lost in *egg*” gene set, regardless of whether SU(VAR)3-9 depletion is caused by *Su(var)3-9* or *egg* mutations, H3K9me2 is altered approximately the same way. This contrasts the situation observed for the “common wt *egg*” gene set. The differences between the mutants are uniformly distributed from −1 to +4 without forming a distinctive peak (Figure S4). Consequently, the contribution of SU(VAR)3-9 and SETDB1 into methylation of these genes is not equal. In the absence of SETDB1, SU(VAR)3-9 binding persists, however the H3K9me2 level drops. Upon SU(VAR)3-9 depletion in *Su(var)3-9* animals, the decrease in H3K9me2 level is far more pronounced.

In order to understand which features are common to SETDB1-dependent targets, wild type SU(VAR)3-9-bound genes were split into three groups, depending on their chromosomal localization, i.e., residing in the euchromatin, heterochromatin, or the chromosome 4. Next, the genes were categorized as either SETDB1-dependent or -independent, and their enrichment at the particular chromosomal location was analyzed (Figure 1a). We observed that SETDB1-dependent genes were predominantly found in the euchromatin, whereas SETDB1-independent genes were significantly more frequent in the heterochromatin and the chromosome 4 (Table S3). We hypothesize that this distribution of SU(VAR)3-9 targets could be related to the distinct sequence composition of eu- vs. heterochromatin. Whereas euchromatin is largely represented by single copy genes, heterochromatin hosts a complex mosaic of various types of repeats. For this reason, SU(VAR)3-9 binding to heterochromatin is primarily repeat-mediated, and so detection of SU(VAR)3-9 in heterochromatic genes is likely associated with their embedding into this unique chromatin environment.

**Figure 3:**
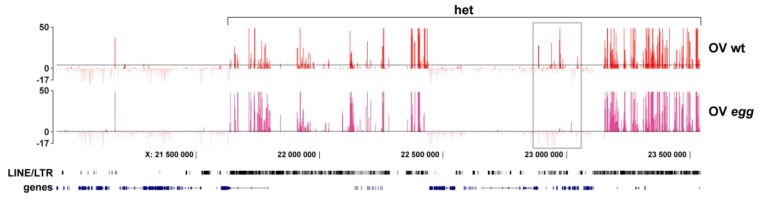
SU(VAR)3-9 profiles for the pericentric heterochromatin of the X chromosome (het) in the germline cells of the wild type (OV wt) and *egg* mutant females (OV *egg*). Single copy heterochromatin-resident genes featuring SETDB1-dependent SU(VAR)3-9 binding are highlighted by the grey frame. The density of grey and black vertical lines on the LINE/LTR panel visualizes the density of various repeated DNA sequences. Axis labels are the same as in Figure 2.

The degree to which SU(VAR)3-9 localization is sensitive to the chromatin context and *egg* mutation is probably best illustrated by the heterochromatin of the X chromosome in germline cells (Figure 3). Distal- and proximal-most parts of this region are dominated by the repeated DNA sequences (LINE/LTR), whereas the middle core is composed of single copy genes. This sequence separation is well correlated with the SU(VAR)3-9 profile, where most of the peaks map to the repeated regions, and few sites are found in the genes. Interestingly, SU(VAR)3-9 binding to repeats is unaffected by *egg*. In contrast, SU(VAR)3-9 is found associated with the genes in the middle part of heterochromatin only in the presence of SETDB1 (grey frame in Figure 3). It must be noted though, that in salivary glands the same region shows rather uniform distribution of SU(VAR)3-9 which is essentially SETDB1-independent (not shown).

**Figure 4:**
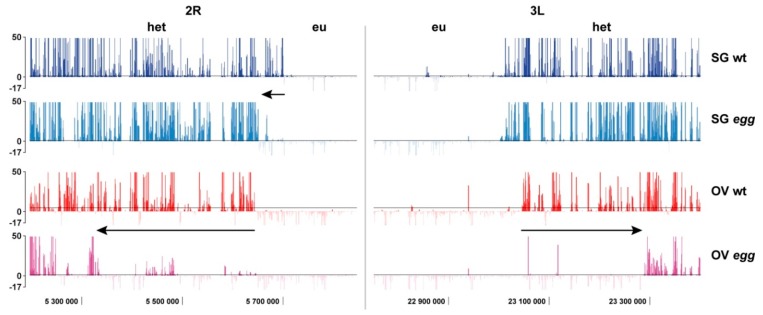
The border between euchromatin (eu) and heterochromatin (het) in the chromosome arms 2R and 3L in the salivary glands (SG) and germline cells of the ovary (OV) from wild type (wt) and *egg* mutants. Data for SG wt are taken from [28], the rest of the profiles are obtained in this study. Axis labels are the same as in Figure 2. Arrows point to the shift between the positions of the border between thegenotypes.

The effects of *egg* mutation on SU(VAR)3-9 binding were also analyzed for the sequences located at the border between eu- and heterochromatin. This border between two chromatin domains is clearly seen as the sharp transition from regions having few if any SU(VAR)3-9 peaks to the regions with high density of peaks in each chromosomal arm and across all the cell types analyzed. Importantly, the position of this border was cell type independent and was unaffected by the meiotic arrest genes *bam*, *aly*, and *can* in *Drosophila* males [28]. In germline cells isolated from juvenile ovaries this border appears a little shifted, compared to the salivary glands (Figure 4, rows 1 and 3). Yet, the effect much more pronounced in *egg* mutant background, and the border moves more proximal. This shift is pronounced to a different extent across the chromosome arms, and is overall stronger in the germline cells, compared to the salivary gland chromatin. For instance, in the chromosome 2R, the shift is ~50 kb for salivary glands, whereas it constitutes ~300 kb in the germline ovarian cells. In the chromosome 3L from salivary glands, the eu/heterochromatin border remains unaffected, whereas it shifts some 200 kb in the germline (Figure 4).

### 3.2. SU(VAR)3-9 Binding to the Chromosomes in the Female Germline is Dynamic throughout Development

**Figure 5:**
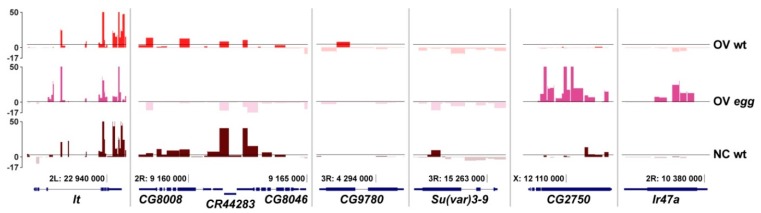
SU(VAR)3-9 binding profiles across select genes depending on the developmental stage of the ovary and the genotype: germline cells of juvenile ovaries of wild type females (OV wt) and *egg* mutants (OV *egg*) or mature nurse cells of wild type (NC wt). Data for NC wt are taken from [28], the rest of the profiles are obtained in this study. Axis labels are the same as in Figure 2.

**Figure 6:**
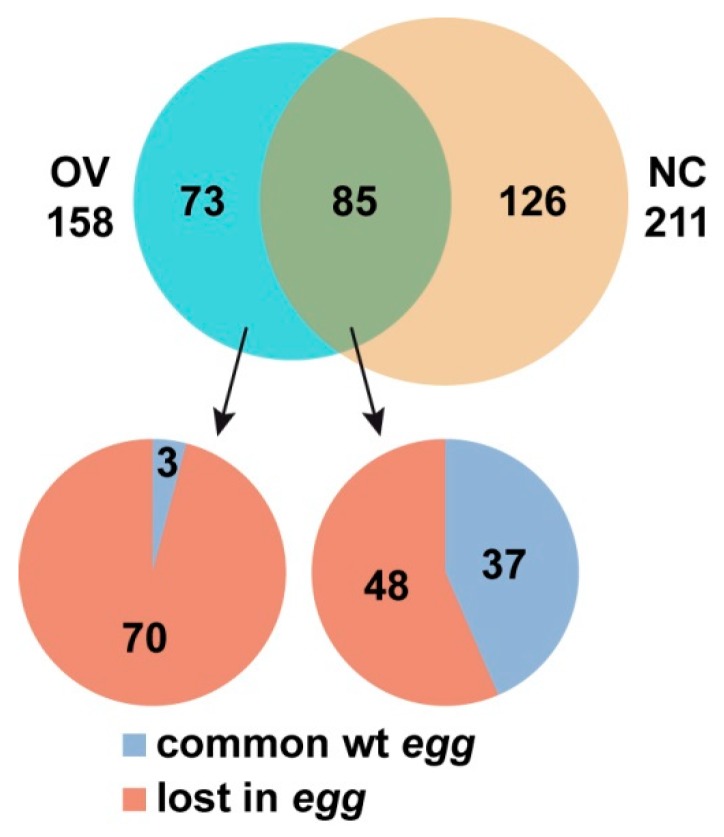
Venn-diagrams showing the numbers of genes that are associated with SU(VAR)3-9 in the ovary in a differentiation-associated manner (upper part); sub-grouping of these gene targets depending on the *egg* effects (bottom part). NC and OV—nurse cells and germline cells of juvenile ovaries, respectively(wild type in both cases). “Common wt *egg*”—gene targets shared between wild type and *egg* profiles. “Lost in *egg*”—genes that are associated with SU(VAR)3-9 only in the presence of SETDB1.

To trace the dynamics of SU(VAR)3-9 binding throughout female germline cell differentiation and to see how this process may be affected by SETDB1, we proceeded to the comparisons of SU(VAR)3-9 profiles at the earliest developmental stage, when the germline tissue is largely composed of the undifferentiated germarium cells (OV wt and OV *egg*), and at a later differentiation stage, i.e., in mature nurse cells of the ovary (NC wt). Several SU(VAR)3-9 binding patterns became apparent (Figure 5). Some genes, such as *lt*, are bound by SU(VAR)3-9 at both differentiation timepoints regardless of the presence of SETDB1. Other examples, such as *CG8008* and nearby genes, are also bound by SU(VAR)3-9 throughout the entire development of the ovary, but this binding is SETDB1-dependent. *CG9780* gene is found associated with SU(VAR)3-9 only in the germline cells of juvenile ovaries, but this binding is progressively reduced throughout differentiation. Interestingly, *Su(var)3-9* gene itself is the target of the SU(VAR)3-9 protein, and this binding is restricted to mature nurse cells, as no SU(VAR)3-9 binding to the *Su(var)3-9* gene is observed in the germarium. Two more unusual patterns of SU(VAR)3-9 distribution are worth noting, exemplified by the genes *CG2750* and *Ir47a*. In both instances, SU(VAR)3-9 is absent from these regions in the juvenile ovaries, unless *egg* mutation is present. Unlike *CG2750* that becomes targeted by SU(VAR)3-9 at a later differentiation stage in the wild type situation, *Ir47a* is not a normal target of SU(VAR)3-9 at any stage of ovarian development. In total, 73 genes were identified whose SU(VAR)3-9 binding was restricted to germarium, 126 genes that are nurse cell-specific, and 85 genes that bind SU(VAR)3-9 at both stages. Thus, the SU(VAR)3-9 profile undergoes differentiation-associated changes in germline cells, and the number of SU(VAR)3-9 targets increases as differentiation proceeds (Figure 6). This is best exemplified by exploring the profiles chromosome-wide (Figure S1). Visual comparison of OV wt vs. NC wt binding profiles indicates that the latter demonstrates far greater SU(VAR)3-9 peak density.

SU(VAR)3-9 distribution pattern appears both stage- and *egg*-dependent. So, our next step was to understand the interplay between these two contributing factors. The two gene target datasets turned out to be related. Specifically, genes that are SU(VAR)3-9-positive exclusively in the germarium, constitute a group that is also nearly always SETDB1-dependent. Of the gene targets that are present regardless of the differentiation stage, the fraction of SETDB1-independent genes is increased (Figure 6, Table S3).

### 3.3. Binding of SU(VAR)3-9 to piRNA Clusters Proceeds via Distinct Mechanisms in the Somatic and Germline Cells

One of the key processes controlled by SETDB1 in the female germline is suppression of transposable elements activity. Multiple piRNA clusters scattered throughout the genome are central to this process [36]. Whereas SETDB1 binding to piRNA clusters has long been known to be required for piRNA precursor synthesis [37], the possible role of SU(VAR)3-9 in this context is far less clear, despite its presence at these loci [28]. Interestingly, transcription at these clusters is not uniformly affected by the *Su(var)3-9* mutation: some targets remain unchanged, some are up- or down-regulated [28]. It was speculated that SU(VAR)3-9 could be required for the spreading of H3K9me within the clusters from the primary methylation site [38].

We focused our analysis of SU(VAR)3-9 distribution across the four large piRNA clusters—38C, 42AB, *flam*, and 20A, —in wild type vs. *egg* mutants, and matched the data with H3K9me2 profiles in wild type, *Su(var)3-9*, and *egg* mutants. Use of H3K9me2 distribution data restricted to salivary glands has been explained above. Clusters of piRNA display different features of organization, activity, and localization. 38C and 42AB map to the autosomal euchromatin, and show dual-strand piRNA expression, whereas 20A and *flam* are found in the heterochromatin of the X chromosome, and display uni-strand expression. Furthermore, expression of the clusters 38C and 42AB is restricted to the germline, *flam* is expressed exclusively in the somatic cells of the ovary, whereas 20A is expressed in both germline and the soma [36].

**Figure 7:**
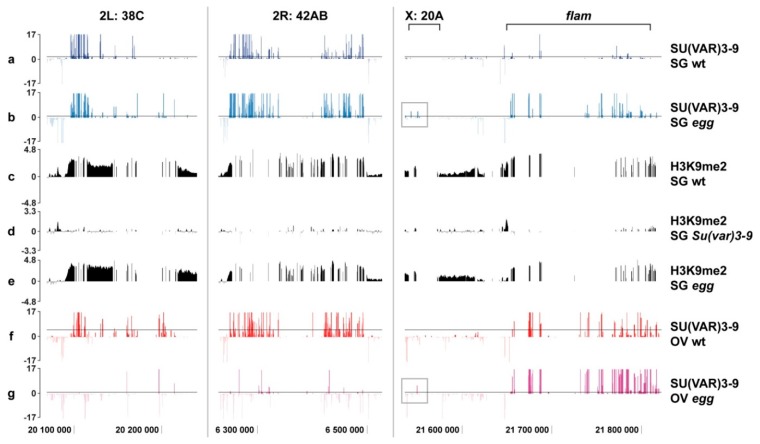
SU(VAR)3-9 distribution (colored profiles) across the four piRNA clusters in salivary glands (SG; **a**,**b**) and germline cells of the juvenile ovaries (OV; **f**,**g**) from wild type (wt) and *egg* mutants, as well as H3K9me2 distribution (black profiles) in the salivary glands from wild type, *egg*, and *Su(var)3-9* mutants (**c**–**e**). Data for SU(VAR)3-9 SG wt are taken from [28]. H3K9me2 profiles have been retrieved from the raw data reported by [35], the rest of the profiles are obtained in the present study. Grey frames in figures (**b**,**g**) denote novel SU(VAR)3-9 peaks in the piRNA cluster 20A, observed in *egg* mutants. Y axis shows significance of SU(VAR)3-9 binding as a −log_10_ scale of *p*-value (colored profiles) or log_2_(IP/input) value for H3K9me2 (black profiles). Genomic coordinates on the X axis correspond to the BDGP Release 6.

In wild type animals, SU(VAR)3-9 associates with the clusters 38C and 42AB in both salivary glands and germline (Figure 7a,f). In *egg* mutants, SU(VAR)3-9 binding remains unchanged in the salivary glands, but is virtually absent in the germline (Figure 7b,g). H3K9me2 is maintained at these clusters in *egg* mutants (Figure 7c,e), but is abolished in *Su(var)3-9* mutants (Figure 7c,d). This is likely attributable to the fact that in the context of salivary glands, SU(VAR)3-9 binding in the piRNA clusters 38C and 42AB is SETDB1-independent. This contrasts the situation observed in the germline, where SETDB1 is required for SU(VAR)3-9 targeting to these clusters.

As for the clusters *flam* and 20A, in the wild type salivary glands and the germline, SU(VAR)3-9 is found associated with *flam* and ignores the 20A (Figure 7a,f). Introducing the *egg* mutant background increases SU(VAR)3-9 binding at these sites in both cell types. Specifically, in the cluster 20A, 1-2 significant binding peaks become visible (grey frames in Figure 7b,g), whereas in the *flam* locus the peak density is higher for salivary glands and less so for the germline (Figure 7b,g). Notably, in the salivary glands, H3K9me2 in both loci is SU(VAR)3-9-dependent and SETDB1-independent (Figure 7c,d,e).

To summarize, one can conclude that piRNA clusters have cell type specific regulation. It is possible that SU(VAR)3-9 binding (with or without SETDB1) and the nature of the protein complex responsible for H3K9 methylation are dependent on their transcriptional activity. Clusters 38C and 42AB are inactive in salivary glands and essentially behave as typical repeated sequences of the pericentric heterochromatin. So, in salivary glands, SU(VAR)3-9 binding and H3K9 methylation are both SETDB1-independent at these sites. In contrast, these clusters have a very specific function in the germline, and so their H3K9 methylation must be compatible with transcription rather than with silencing. This is now under the control of a distinct enzyme, SETDB1, and SU(VAR)3-9 has a downstream function of spreading of the methylation mark. The same logic can be applied to explain the SU(VAR)3-9 binding at the *flam* locus. It is only active in the somatic cells of the ovary, and is inactive in the germline cells and salivary glands, thereby blending in with the surrounding heterochromatin. This locus is SU(VAR)3-9-positive in both cells types, and this binding is weakly affected by the *egg* mutation. Cluster 20A is active in the germline, however, unlike 38C and 42AB, it has uni-strand expression, and so it is under a distinct mechanism of transcription control [39]. This may explain the lack of appreciable SU(VAR)3-9 binding as well as a very weak effect of the *egg* mutation at the 20A, which is opposite to the effect observed at the clusters 38C and 42AB.

## 4. Discussion

### 4.1. Interplay between SU(VAR)3-9 and SETDB1

In this work, we continued our earlier efforts to analyze SU(VAR)3-9 distribution on the chromosomes from different *Drosophila* tissues and demonstrated the important role of SETDB1 in binding of SU(VAR)3-9 to its targets. Previously, we speculated that SU(VAR)3-9 may have a context-dependent role. In euchromatin, it may be required for fine-tuning the expression of select genes, whereas in heterochromatin, piRNA clusters and the chromosome 4 it rather acts as a domain-wide regulator [28]. Our present findings indicate that the mechanisms of SU(VAR)3-9 recruitment to chromatin may differ in different chromosomal contexts. In euchromatic single copy genes, the presence of SU(VAR)3-9 is largely SETDB1-dependent, whereas heterochromatic repeated sequences bind SU(VAR)3-9 largely independent of SETDB1. This trend is generally applicable for both somatic and germline cells. Similar context dependency of SU(VAR)3-9 binding has been recently described for *C. elegans*, where SET-25 (SU(VAR)3-9 ortholog) is responsible for H3K9 tri-methylation in retrotransposons and pseudogenes, whereas MET-2 (SETDB1 ortholog) controls H3K9 di-methylation in the satellites [40].

Profound differences between somatic and germline cells are observed for the chromosome 4 and piRNA clusters, which is likely attributable to the peculiar functions of these genomic regions. Chromosome 4 displays massive SU(VAR)3-9 enrichment in salivary glands, but not in other cell types analyzed [28]. In *egg* mutants, SU(VAR)3-9 binding over the chromosome 4 decreases, but given the initial strong enrichment, most of the SU(VAR)3-9 targets remain unaffected and continue to bind SU(VAR)3-9. This is unlike the situation observed for the chromosome 4 in the germline, where SU(VAR)3-9 targets are no longer detectable upon depletion of SETDB1 (see Figure 1a and Figure S1). As for piRNA clusters, their expression is largely restricted to the germline, and absent in the salivary glands, which may explain the discordant SU(VAR)3-9 binding in these tissues (see above).

Exactly how SETDB1 influences SU(VAR)3-9 recruitment to its targets, remains an open question. Several options can be imagined. One could be similar to the mechanism described for piRNA clusters, where SU(VAR)3-9 operates to spread the methyl mark [38]. In this case, SETDB1 may di-methylate H3K9 thereby creating the substrate recognized by the chromodomain of SU(VAR)3-9, which in turn maintains this methylation mark. This scenario is supported by the fact that SETDB1 is the key enzyme producing H3K9me2 in the pericentric heterochromatin in early *Drosophila* embryos, and the subsequent maintenance step is controlled by SU(VAR)3-9 [41]. The second option relates to the existence of a common protein complex encompassing SETDB1 and SU(VAR)3-9 similar to the one described in mammals [30]. In this case, lack of SETDB1 may prevent the complex from forming and block SU(VAR)3-9 binding to its targets. Given that our work used *egg^1473^* allele characterized by the in-frame deletion of the entire SET domain [22], one can speculate that it is histone methyltransferase activity of SETDB1 that is required for SU(VAR)3-9 targeting.

SU(VAR)3-9 binding is abrogated for most genomic targets in the *egg* mutant background. Nonetheless, several novel SU(VAR)3-9 binding sites have been observed to appear as well. Upon closer inspection of this group of sites, several possible reasons why this may occur became apparent. First, our data processing algorithm allows for a 5% FDR, therefore, some of these novel SU(VAR)3-9 targets in *egg* mutants could be the result of statistical error. Second, most of the heterochromatin-embedded genes are known to have short exons and large introns hosting repeated sequences. In such heterochromatic genes, SU(VAR)3-9 is typically found associated with introns rather than exons. In *egg* mutants, a certain degree of SU(VAR)3-9 spreading may occur, and so the intron-mapping peak may “touch” the adjacent exon. This in turn results in classifying this gene as a novel target, but in fact is a mere consequence of its localization in the heterochromatin. Finally, the third reason may be related to the intrinsic peculiarities of these genes, that truly become targeted by SU(VAR)3-9 upon depletion of SETDB1, as exemplified by the gene *Ir47a* (see Figure 5) as well as the genes such as *sls* showing intragenic re-localization of SU(VAR)3-9 (see Figure 2 and Figure S3). It may well be that SU(VAR)3-9 and SETDB1 may function interchangeably, which could explain why SU(VAR)3-9 appears in *egg* mutants at the regions that are normally occupied by SETDB1. This intriguing option is a matter of future research, and will be analyzed by exploring SETDB1 binding profiles across these gene subsets.

Changes in SU(VAR)3-9 binding are expected to correlate with altered H3K9me2 levels, and our analyses confirm this is indeed the case. Reduced and increased methylation of SETDB1-dependent genes is directly associated with the loss and acquisition of SU(VAR)3-9 in *egg* background, respectively. It must be underlined, that this trend is merely a correlation rather than a dependence. Figure 1b illustrates that in *egg* mutants approximately 20% of “lost in *egg*” genes have a positive value of H3K9 methylation change, indicating that the disappearance of SU(VAR)3-9 has led to increased H3K9 methylation.

By performing pairwise comparisons (*egg* vs. *Su(var)3-9* mutants) of the box-plots for “lost in *egg*” and “common wt *egg*” gene sets (see Figure 1b and Figure S4), one can see that these sets differ not only in how *egg* mutation affects SU(VAR)3-9 binding but also in the relative contribution of the methyltransferases into H3K9 methylation. Genes belonging to the “lost in *egg*” category lose SU(VAR)3-9 in both *egg* and *Su(var)3-9* mutants. It is unclear whether these genes are normally associated with SETDB1 but in *egg* mutants neither SETDB1 nor SU(VAR)3-9 are found at these genes. By definition, in *Su(var)3-9* animals, these genes are not targeted by SU(VAR)3-9, and the status of SETDB1 binding is not known. Nonetheless, in both mutants the level of H3K9me2 is shifted approximately to the same extent. Consequently, H3K9 methylation of genes from the “lost in *egg*” gene set is either primarily controlled by SU(VAR)3-9, or both enzymes contribute equally. This is unlike the situation observed in the “common wt *egg*” gene set. Here, in the absence of SETDB1, SU(VAR)3-9 persists, yet this results in lower levels of H3K9 methylation. Hence, this methylation is in all likelihood mediated by SETDB1. In *Su(var)3-9* mutants, these genes lack SU(VAR)3-9, the status of SETDB1 association is unknown, and H3K9 methylation level decreases even further. We believe that “common wt *egg*” genes constitute a category wherein histone methylation is dependent on both proteins, more on SU(VAR)3-9, and less so on SETDB1. Unfortunately, our speculations are limited by the lack of SETDB1 distribution data, and more experiments are needed to uncover the relative contribution of each enzyme into H3K9 methylation.

H3K9me2 pattern in the “new in *egg*” set is quite peculiar. These genes are not associated with SU(VAR)3-9 in wild type, yet become SU(VAR)3-9-bound in *egg* mutants, which predictably leads to up-regulated H3K9 methylation. However, the amount of H3K9me2 drops in *Su(var)3-9* background for these genes, contrary to the expectation that it would remain unaltered. This small gene set is formed by a composite mix of genes, as described above, and each gene in this category may have its own regulation pattern without a common trend.

Our previous report demonstrated that the coding parts of the genes are significantly enriched and introns are significantly depleted for SU(VAR)3-9 in the context of euchromatin-residing genes. Furthermore, moderate, yet significant SU(VAR)3-9 enrichment is also observed for the alternatively spliced genes [28]. In human cells, the presence of H3K9me2/3 mark in the alternative exons was reported to serve as the signal for their inclusion into the mRNA [12,13]. By drawing these parallels, one may expect that SU(VAR)3-9 may have a role in alternative splicing. Given that the presence of SU(VAR)3-9 in alternative exons may be *egg*-dependent, SETDB1 may also be involved in this regulation. Presently, the details of SETDB1 distribution within genes are not known and it is unclear whether it has a certain predilection to alternative exons. Indirect evidence indicating this may be the case exists. For instance, in the context of chromosome 4 in *Drosophila*, SETDB1 is physically associated with the protein POF, and POF association with genes where the two proteins co-localize is known to be SETDB1-dependent [20,29]. Notably, POF binding is strongly biased towards exon sequences [42]. Also, in human cells, the 3′-exons of many zinc finger genes were reported to be specifically covered by H3K9me3 [43], where this histone mark co-localizes with SETDB1 [44].

### 4.2. SU(VAR)3-9 and SETDB1 in the Female Germline Cells

Both SU(VAR)3-9 and SETDB1 are active in the female germline and function sequentially throughout differentiation, with maximum *egg* and *Su(var)3-9* mRNA levels found in the germarium and mature egg chambers, respectively [21]. We hypothesize that the SU(VAR)3-9 binding profiles observed in the germline cells, as well as the *egg*- and differentiation-associated changes can be attributed to the differential activity of the underlying genes. *Su(var)3-9* mRNA levels increase significantly throughout differentiation, and so does the number of SU(VAR)3-9 targets. At the same time, *Su(var)3-9* mRNA levels in the germarium are relatively low, yet sufficient for the strong recruitment of SU(VAR)3-9 to the pericentric heterochromatin. We observed that the vast majority of targets whose association with SU(VAR)3-9 is restricted to the germarium (i.e., the tissue having top *egg* mRNA levels) are SETDB1-dependent, and their percentage drops as differentiation proceeds (see Figure 6). Apparently, germline development is accompanied not only with the replacement of one H3K9-specific histone methyltransferase by another one, but also with the overall re-wiring of gene regulation. In the germarium, SU(VAR)3-9 acts predominantly via a SETDB1-dependent mechanism, whereas by the end of the germline development it functions essentially as a standalone protein regardless of the presence of SETDB1.

### 4.3. Border between Eu- and Heterochromatin

The topics of where the molecular border delimiting eu- and heterochromatin is located, whether it is permanent and, if not, how it is controlled have been a subject of active research in the past. Sharp changes in H3K9me2 levels are associated with the transition from eu- to heterochromatin. These changes are clearly related to the ratio of unique vs. repeated DNA sequences, as both repeats and H3K9me2 are increased at the border [45]. Intriguingly, the “epigenetic” border between eu- and heterochromatin was shown to be labile and shift several hundred kb in different cell types [46] despite the identical DNA composition. The position of the border may also be controlled by the balance between the antagonistic chromatin modifying proteins. For instance, both the hypermorphic mutant *Su(var)3-9^ptn^* and *Su(var)3-9* overexpression result in massive heterochromatinization, whereas in *JIL-1^Su(var)3-1^* hypermorphic mutants heterochromatin expansion is repressed [18]. In the present study, we observed that the position of eu/heterochromatin border is influenced by SETDB1. This observation is in line with the idea on the pivotal role of the balance between chromatin modifiers, rather than the DNA sequence composition, in setting the border between these two types of chromatin.

## Supplementary Materials

The following are available online at https://www.mdpi.com/2073-4409/8/9/1030/s1, Figure S1: General view of SU(VAR)3-9 binding profiles in the chromosomes from all the samples. SG—salivary glands, OV—juvenile ovaries (mainly consisting of germarium cells), NC—mature nurse cells, wt—wild type, *egg*—*eggless* mutation. Data for SG *egg*, OV wt, and OV *egg* are obtained in the present study, data for SG wt and NC wt are taken from [28]. PH—pericentric heterochromatin. Axis labels are the same as in Figure 2. Figure S2: SU(VAR)3-9 binding (colored profiles) and H3K9me2 distribution (black profiles) in the region 31B-E of the chromosome arm 2L in salivary glands (SG) of wild type (wt), *egg*, and *Su(var)3-9* mutant larvae. SU(VAR)3-9 SG wt profile is taken from [28]. H3K9me2 profiles have been retrieved from the raw data reported by [35]. Axis labels are the same as in Figure 7. Figure S3: SU(VAR)3-9 binding profiles across the genes *trol*, *rg*, and *SK* in the salivary glands (SG) of wild type (wt) and *egg* mutant larvae. SU(VAR)3-9 SG wt profile is taken from [28]. Axis labels are the same as in Figure 2. Figure S4: Distribution of gene numbers in “lost in *egg*” and “common wt *egg*” gene sets, based on the relative contribution of *Su(var)3-9* vs. *egg* to H3K9me2 levels. For each gene, the difference between *egg* and wild type values, as well as between *Su(var)3-9* and wild type values are calculated as log ratio. The latter is subtracted from the former. X axis shows the resulting values (in increments of 0.5), Y axis shows the number of genes in each group (N). Table S1: Correlations between technical and biological replicates in the sequencing data. SG wt—larval salivary glands (female), wild type; SG *egg*—larval salivary glands (female), *egg* mutants; OV wt—germline cells from juvenile ovaries of wild type females; OV *egg*—germline cells from *egg* ovaries; NC wt—nurse cells, wild type. SG wt and NC wt data are taken from [28]. Table S2: Lists of genes that display significant SU(VAR)3-9 binding. SG wt—larval salivary glands (female), wild type; SG *egg*—larval salivary glands (female), *egg* mutants; OV wt—germline cells from juvenile ovaries of wild type females; OV *egg*—germline cells from *egg* ovaries; NC wt—nurse cells, wild type. Table S3: (**a**) Assessment of *p*-values for the Figure 1 and Figure 6. We calculated the significance of differences between the pairs of samplings shown in the bottom row of images. For instance, Figure 1 features total of 413 “salivary glands wt only” genes, for which the ratio of eu:het:chr 4 gene targets is 374:16:23. Consequently, for the sampling “salivary glands wt *egg* common” (a total of 216 genes) the expected ratio should be 196:8:12, yet the observed ratio is 93:72:51. Similarly, the significances of differences were calculated for the germline cells in Figure 1, as well as for the samplings shown in Figure 6. (**b**) Significance of differences (*p*-values) between the groups of genes and mutants shown in Figure 1b.
