# Supplementary material for: Binding of SU(VAR)3-9 Partially Depends on SETDB1 in the Chromosomes of Drosophila melanogaster
Source: Cells. 2019 Sep 5;8(9):1030. doi: 10.3390/cells8091030 (PMC6769583; doi:10.3390/cells8091030)
Supplement: Supplementary file 1 [file cells-08-01030-s001.zip › cells-562780-supplementary/cells-562780_Supplementary_material.pdf]

Supplementary materials

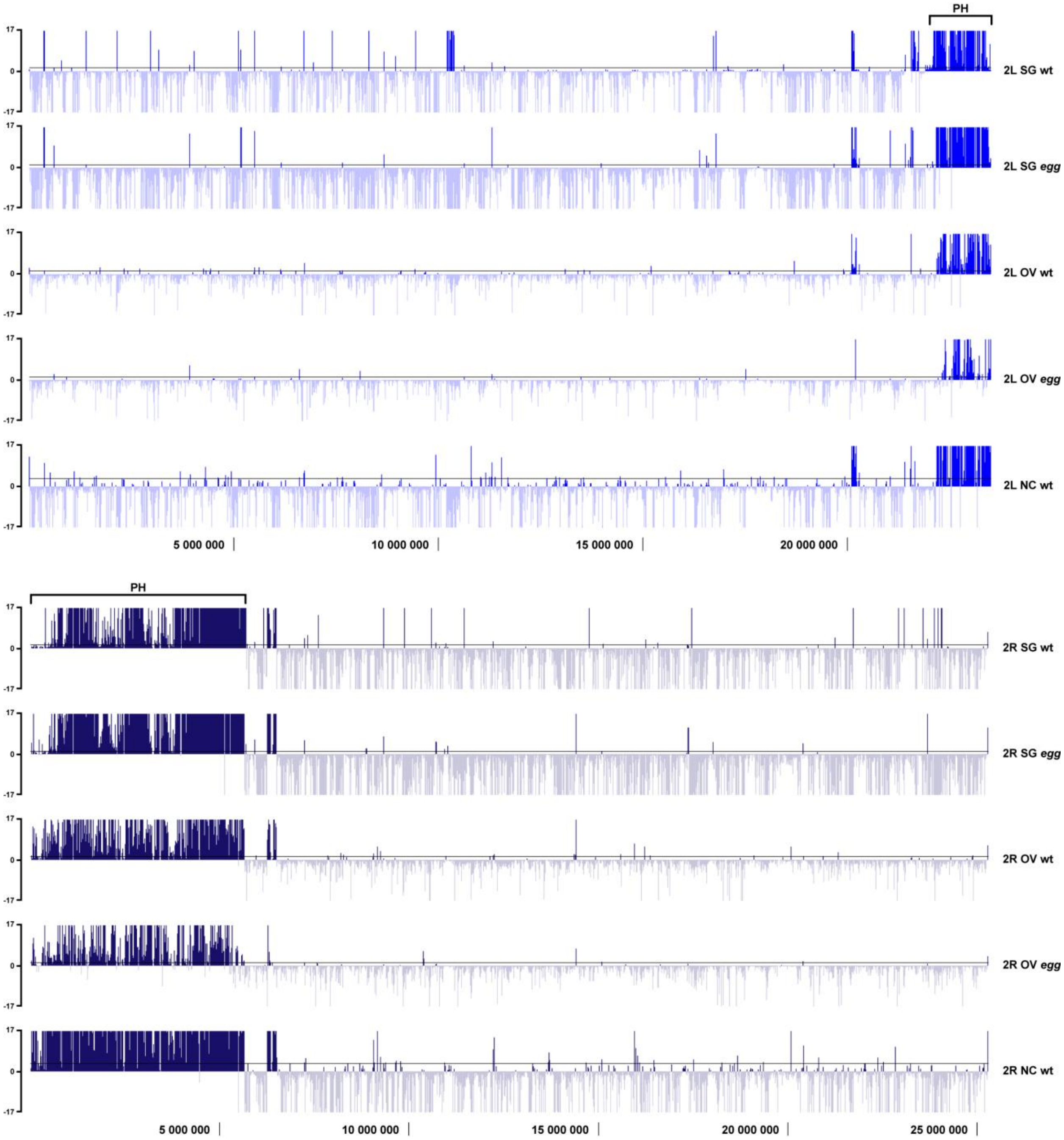

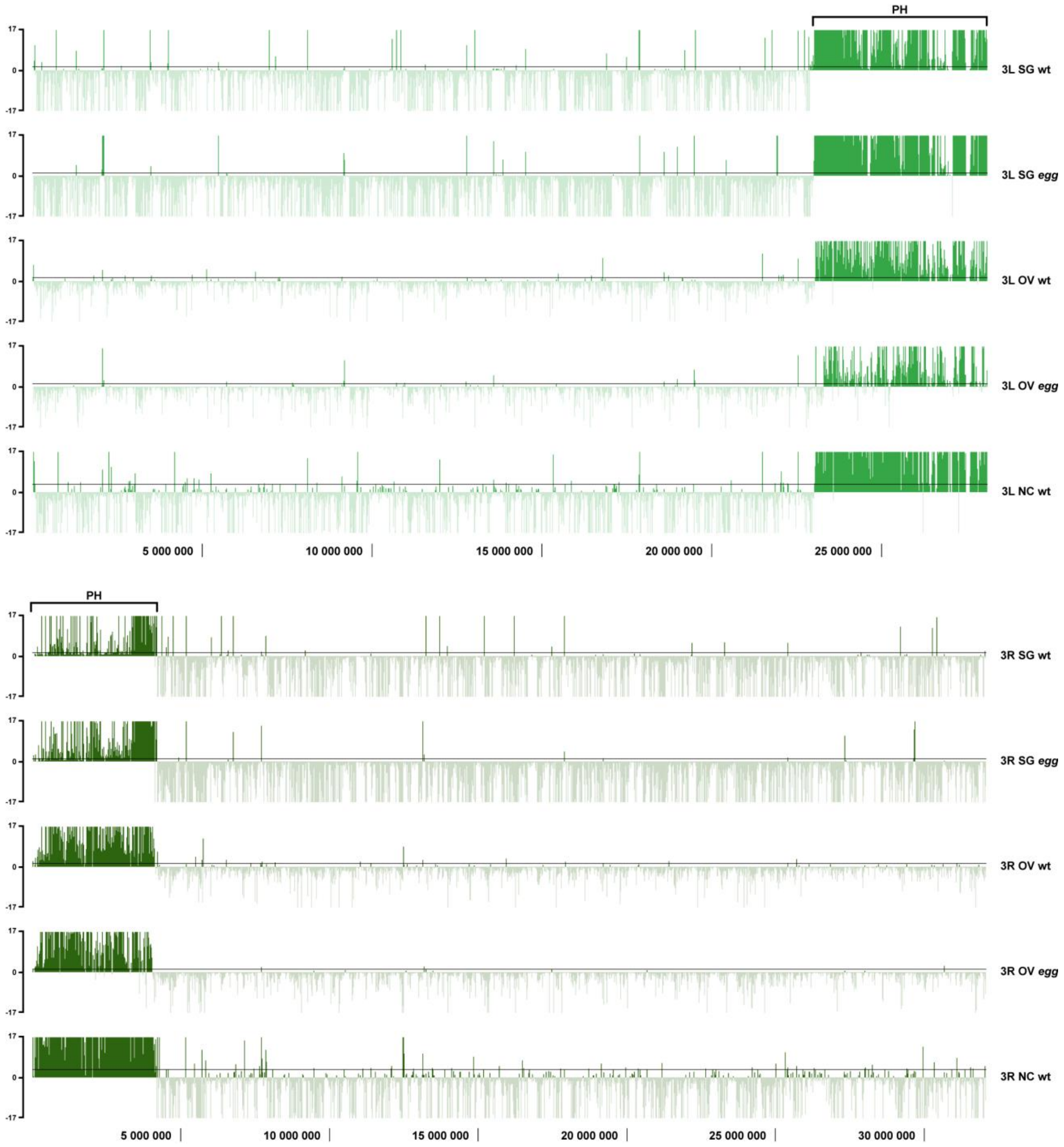

PH
17
0
-17
3L SG wt
3L SG *egg*
3L OV wt
3L OV *egg*
3L NC wt
5 000 000
10 000 000
15 000 000
20 000 000
25 000 000
PH
3R SG wt
3R SG *egg*
3R OV wt
3R OV *egg*
3R NC wt
5 000 000
10 000 000
15 000 000
20 000 000
25 000 000
30 000 000

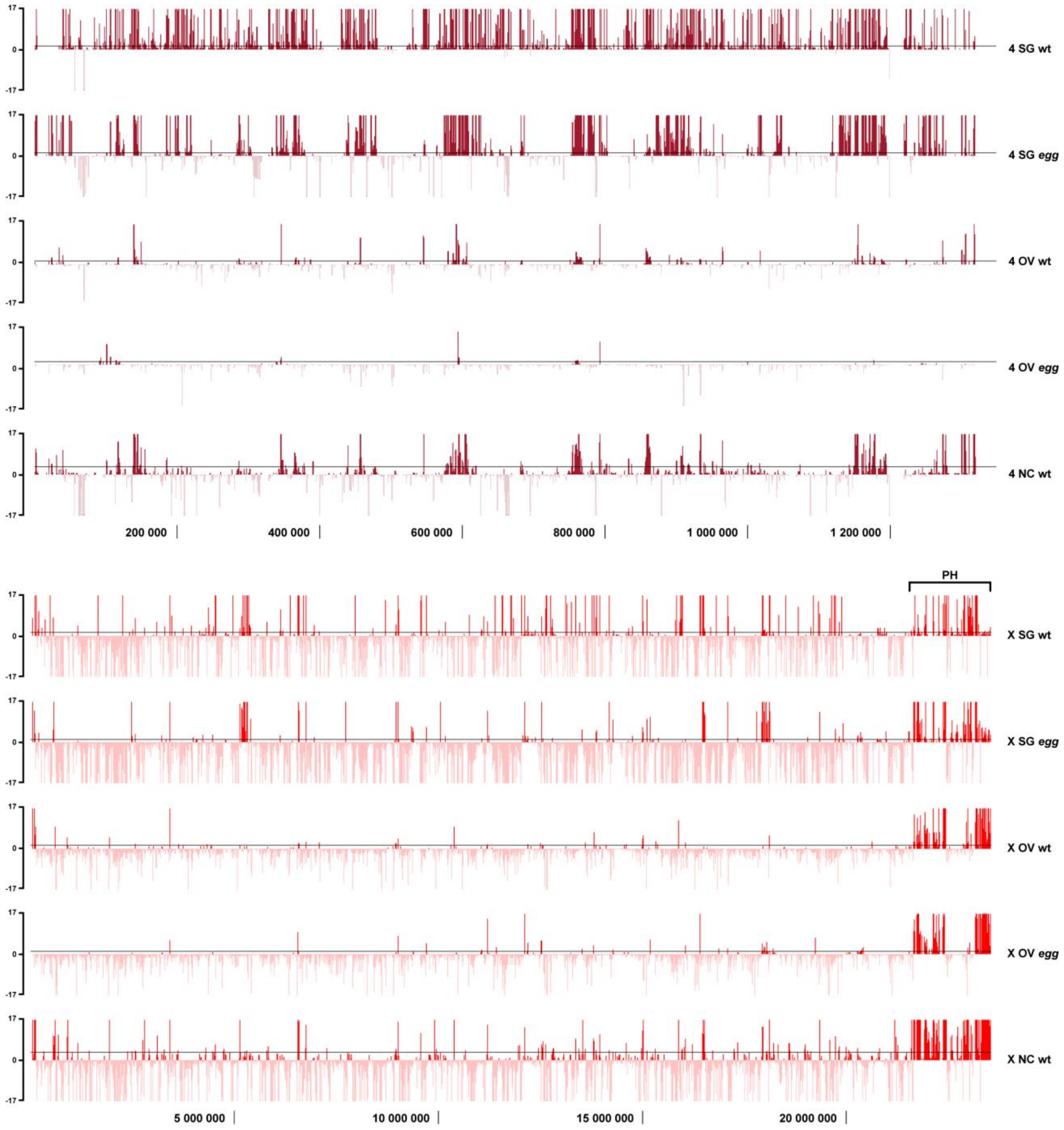

Figure 1. General view of SU(VAR)3-9 binding profiles in the chromosomes from all the samples. SG – salivary glands, OV – juvenile ovaries (mainly consisting of germarium cells), NC – mature nurse cells, wt – wild type, *egg* – *eggless* mutation. Data for SG *egg*, OV wt, and OV *egg* are obtained in the present study, data for SG wt and NC wt are taken from [28]. PH – pericentric heterochromatin. Axis labels are the same as in **Figure 2**.

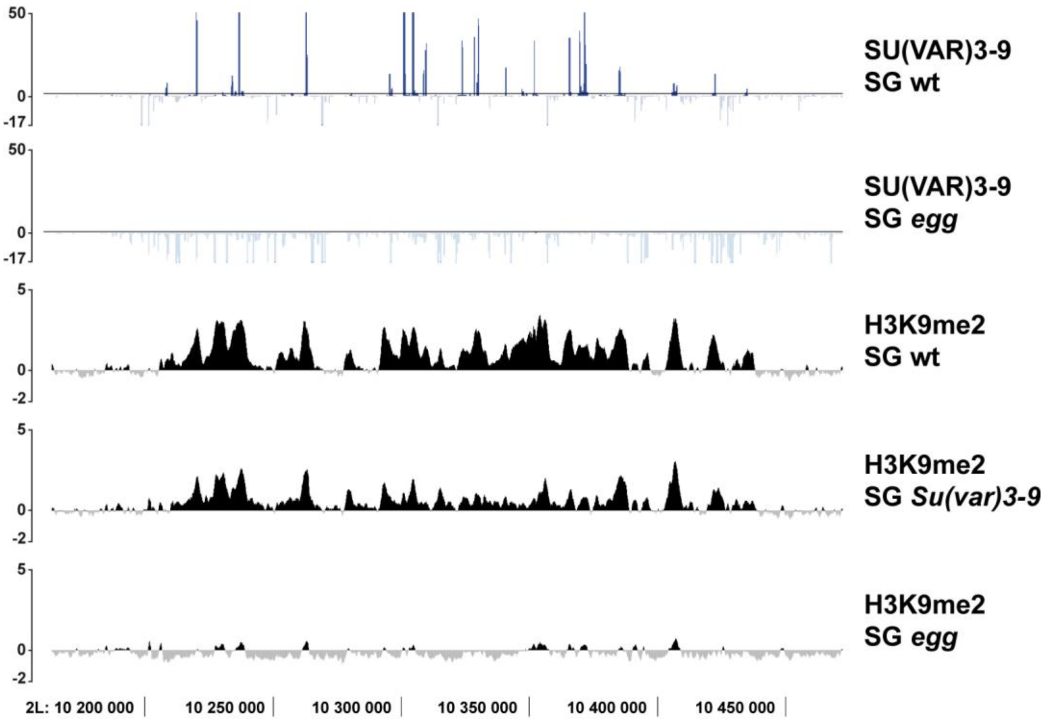

Figure S2. SU(VAR)3-9 binding (colored profiles) and H3K9me2 distribution (black profiles) in the region 31B-E of the chromosome arm 2L in salivary glands (SG) of wild type (wt), *egg*, and *Su(var)3-9* mutant larvae. SU(VAR)3-9 SG wt profile is taken from [28]. H3K9me2 profiles have been retrieved from the raw data reported by [35]. Axis labels are the same as in **Figure 7**.

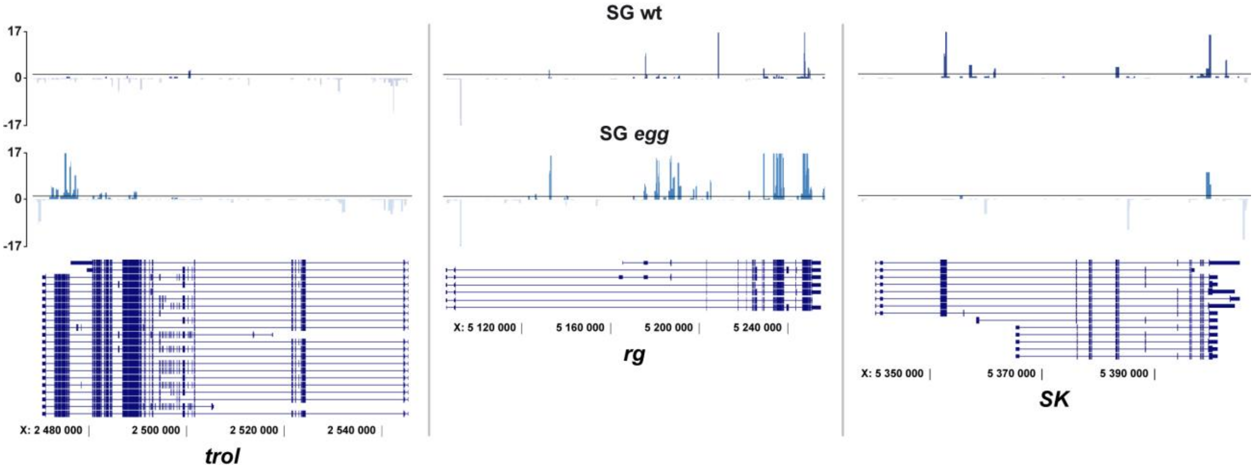

Figure S3. SU(VAR)3-9 binding profiles across the genes *trol*, *rg*, and *SK* in the salivary glands (SG) of wild type (wt) and *egg* mutant larvae. SU(VAR)3-9 SG wt profile is taken from [28]. Axis labels are the same as in **Figure 2**.

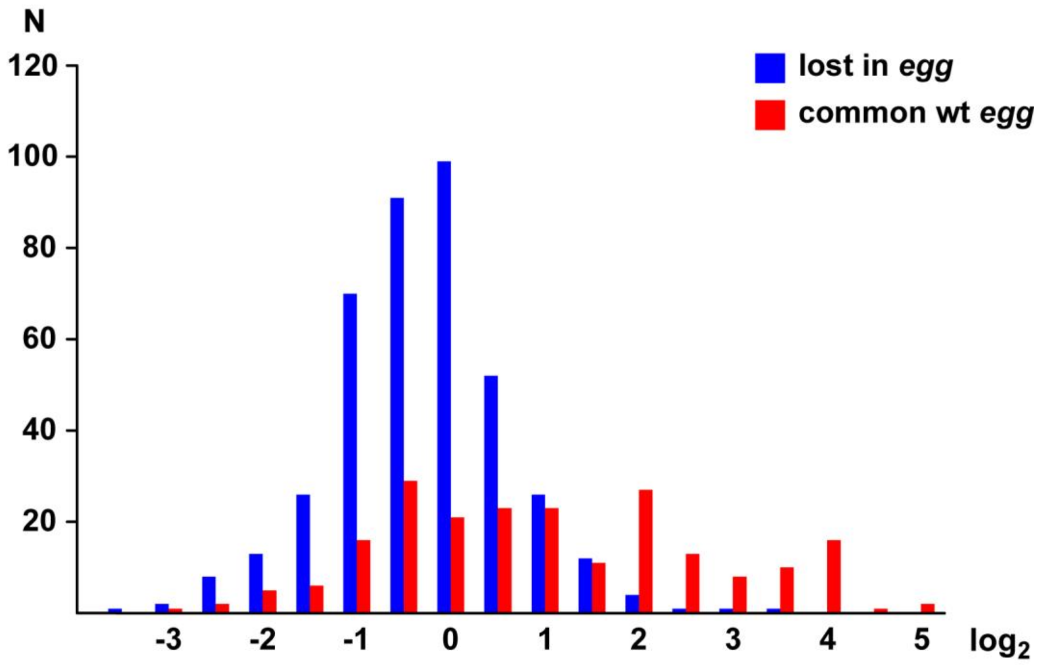

Figure S4. Distribution of gene numbers in "lost in *egg*" and "common wt *egg*" gene sets, based on the relative contribution of *Su(var)3-9* vs *egg* to H3K9me2 levels. For each gene, the difference between *egg* and wild type values, as well as between *Su(var)3-9* and wild type values are calculated as log ratio. The latter is subtracted from the former. X axis shows the resulting values (in increments of 0.5), Y axis shows the number of genes in each group (N).
