# Supplementary figures and images for: Binding of SU(VAR)3-9 Partially Depends on SETDB1 in the Chromosomes of Drosophila melanogaster
Source: Cells. 2019 Sep 5;8(9):1030. doi: 10.3390/cells8091030 (PMC6769583; doi:10.3390/cells8091030)

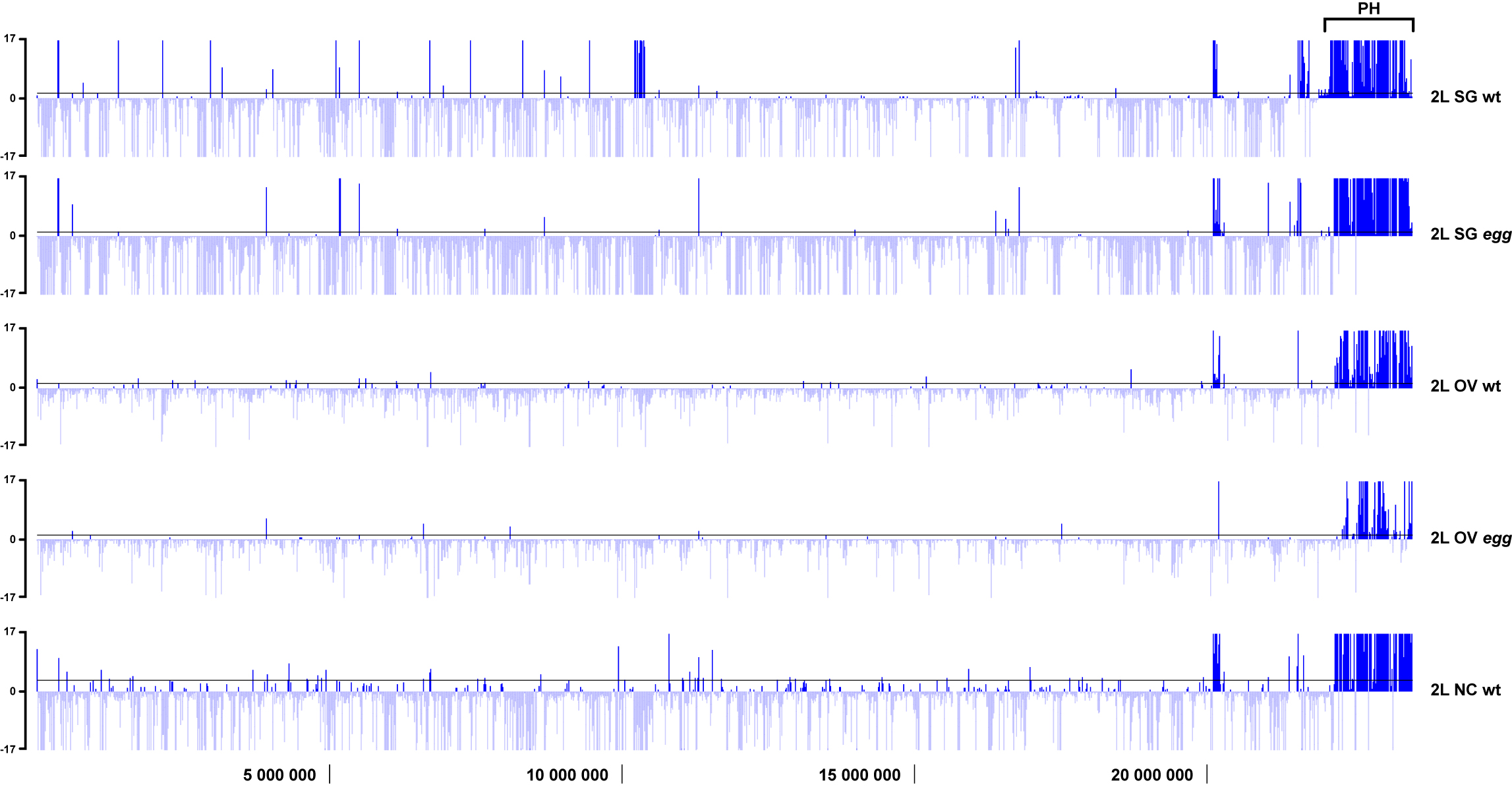

Supplement: Supplementary file 1 [file cells-08-01030-s001.zip › cells-562780-supplementary/cells-562780_Fig_S1_chr2l.jpg]

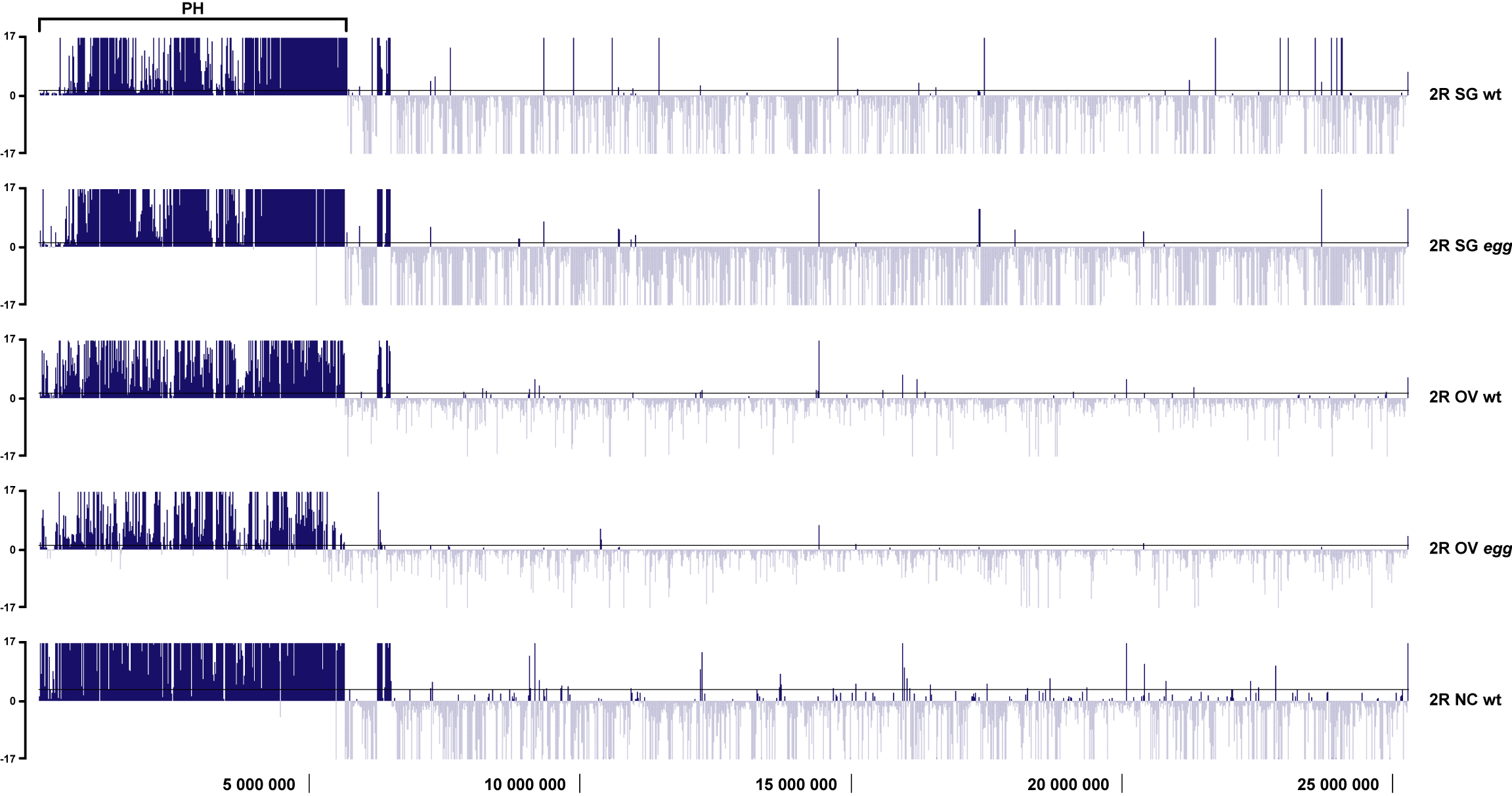

Supplement: Supplementary file 1 [file cells-08-01030-s001.zip › cells-562780-supplementary/cells-562780_Fig_S1_chr2r.jpg]

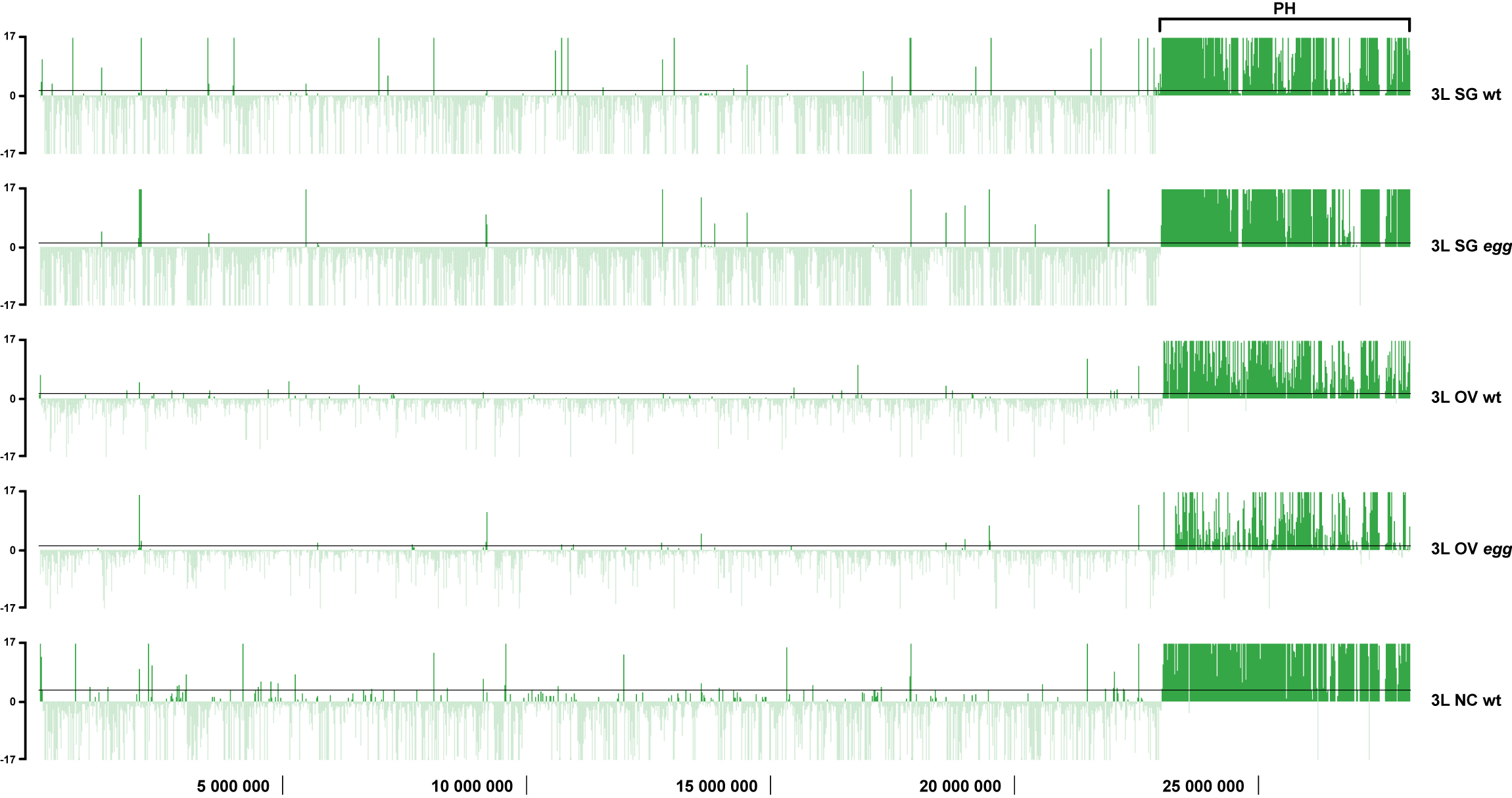

Supplement: Supplementary file 1 [file cells-08-01030-s001.zip › cells-562780-supplementary/cells-562780_Fig_S1_chr3l.jpg]

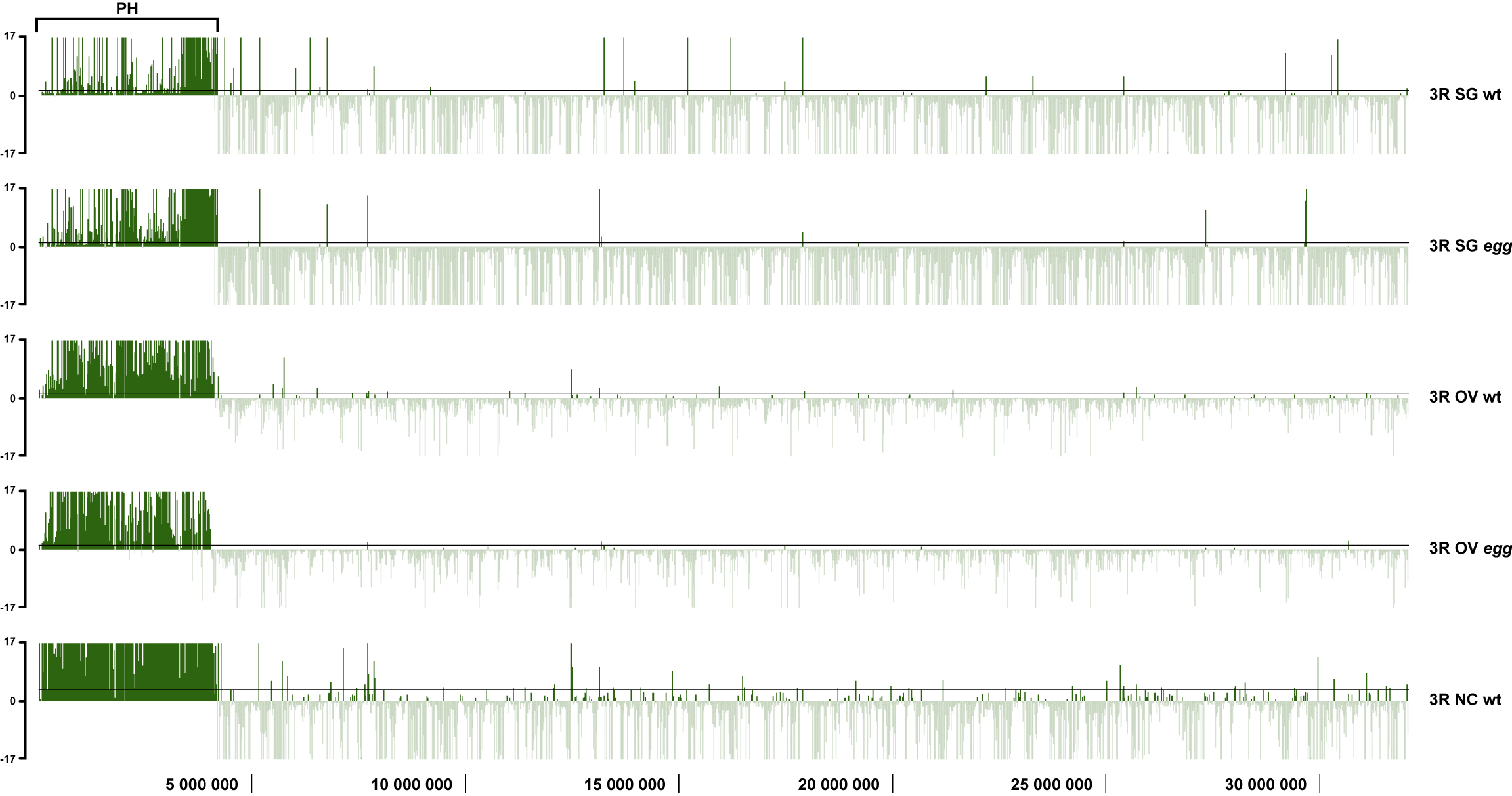

Supplement: Supplementary file 1 [file cells-08-01030-s001.zip › cells-562780-supplementary/cells-562780_Fig_S1_chr3r.jpg]

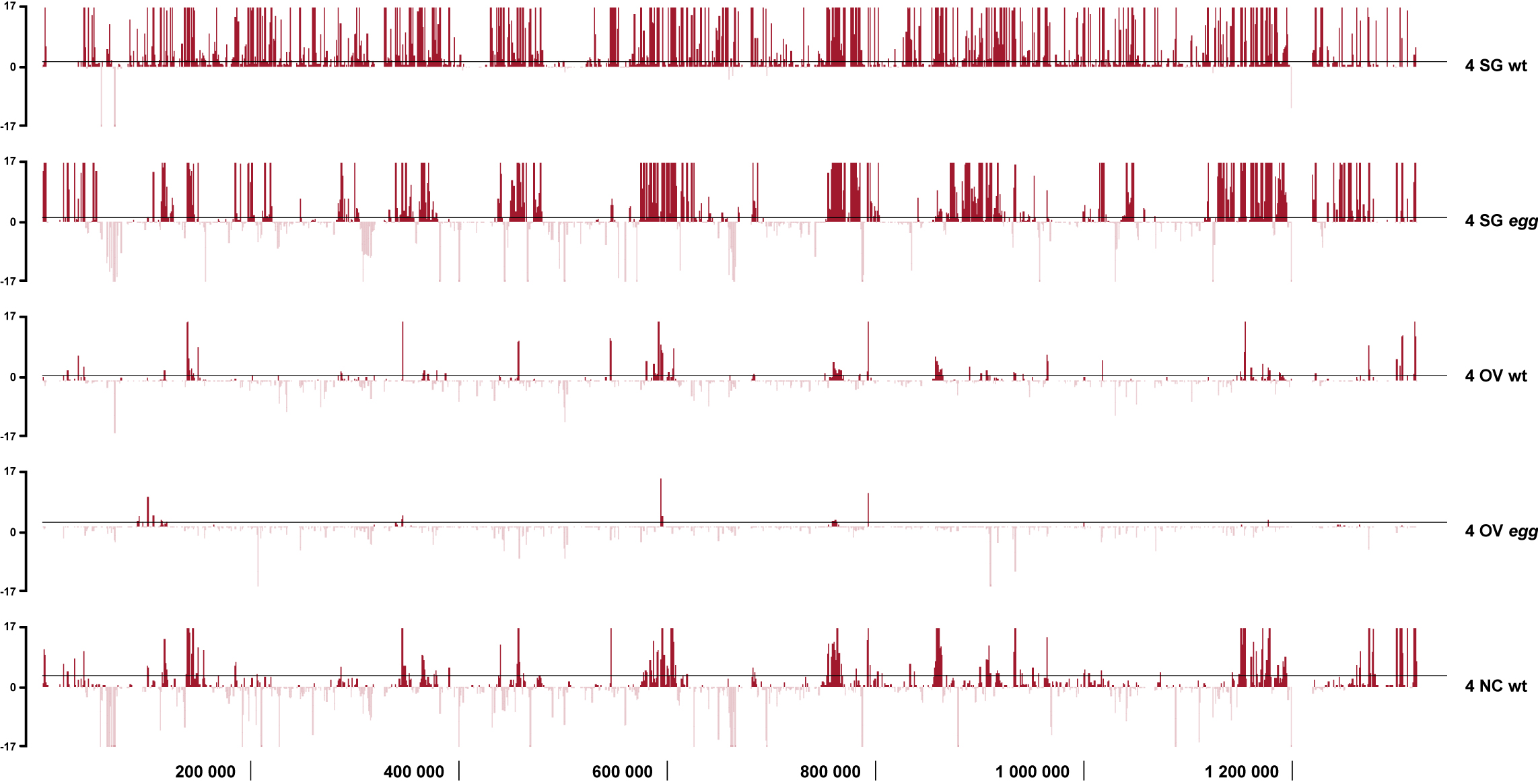

Supplement: Supplementary file 1 [file cells-08-01030-s001.zip › cells-562780-supplementary/cells-562780_Fig_S1_chr4.jpg]

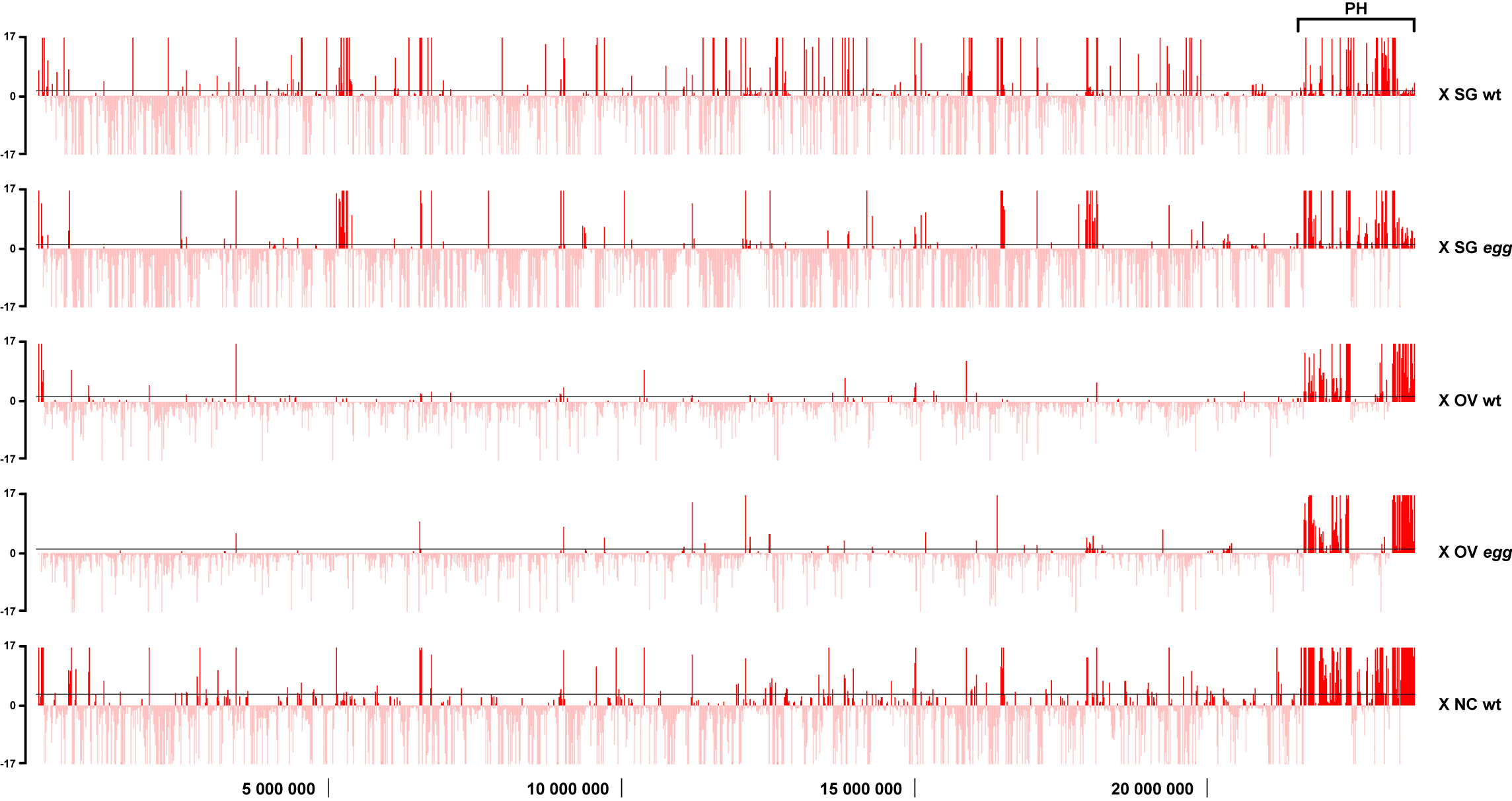

Supplement: Supplementary file 1 [file cells-08-01030-s001.zip › cells-562780-supplementary/cells-562780_Fig_S1_chrX.jpg]

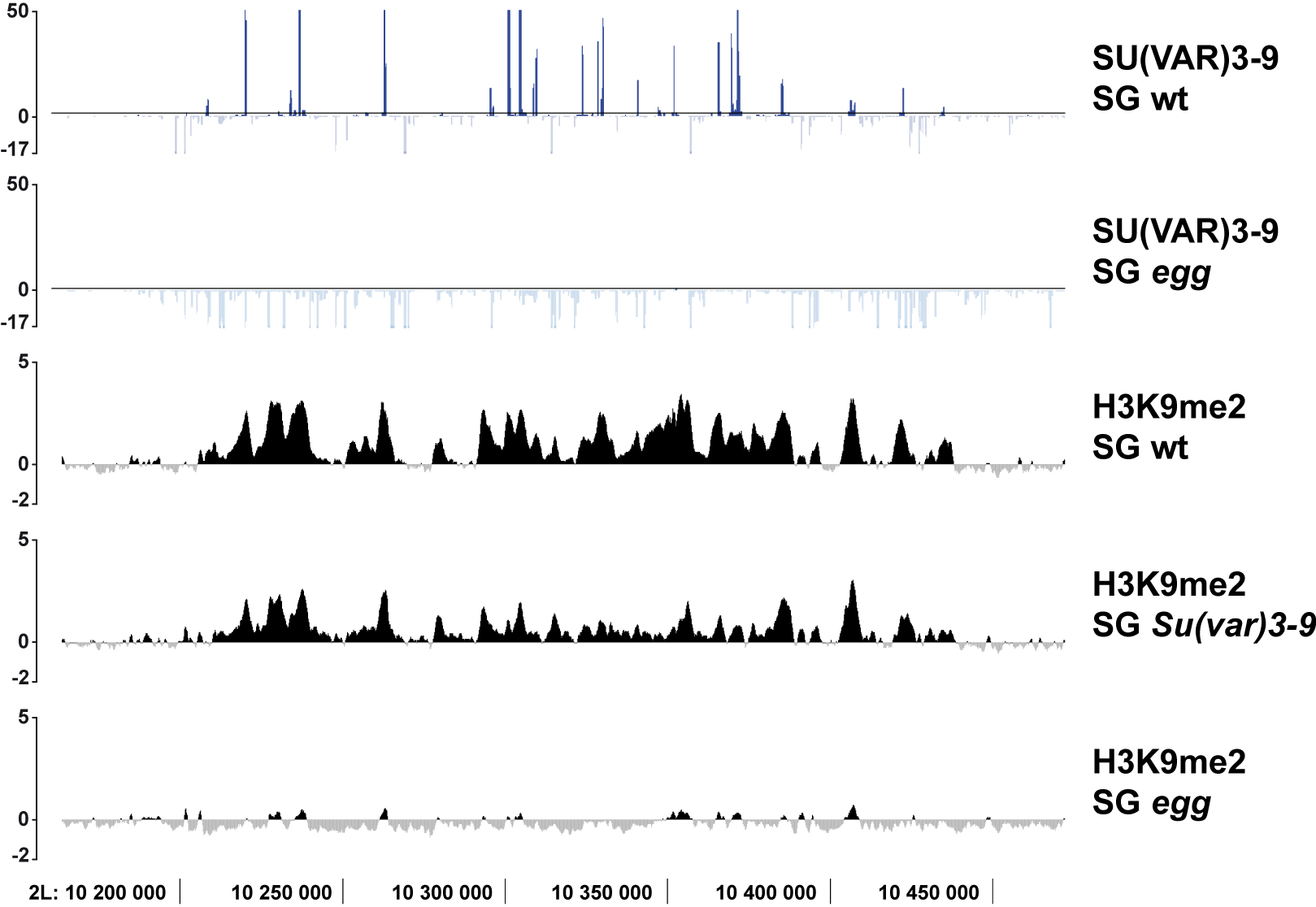

Supplement: Supplementary file 1 [file cells-08-01030-s001.zip › cells-562780-supplementary/cells-562780_Fig_S2.jpg]

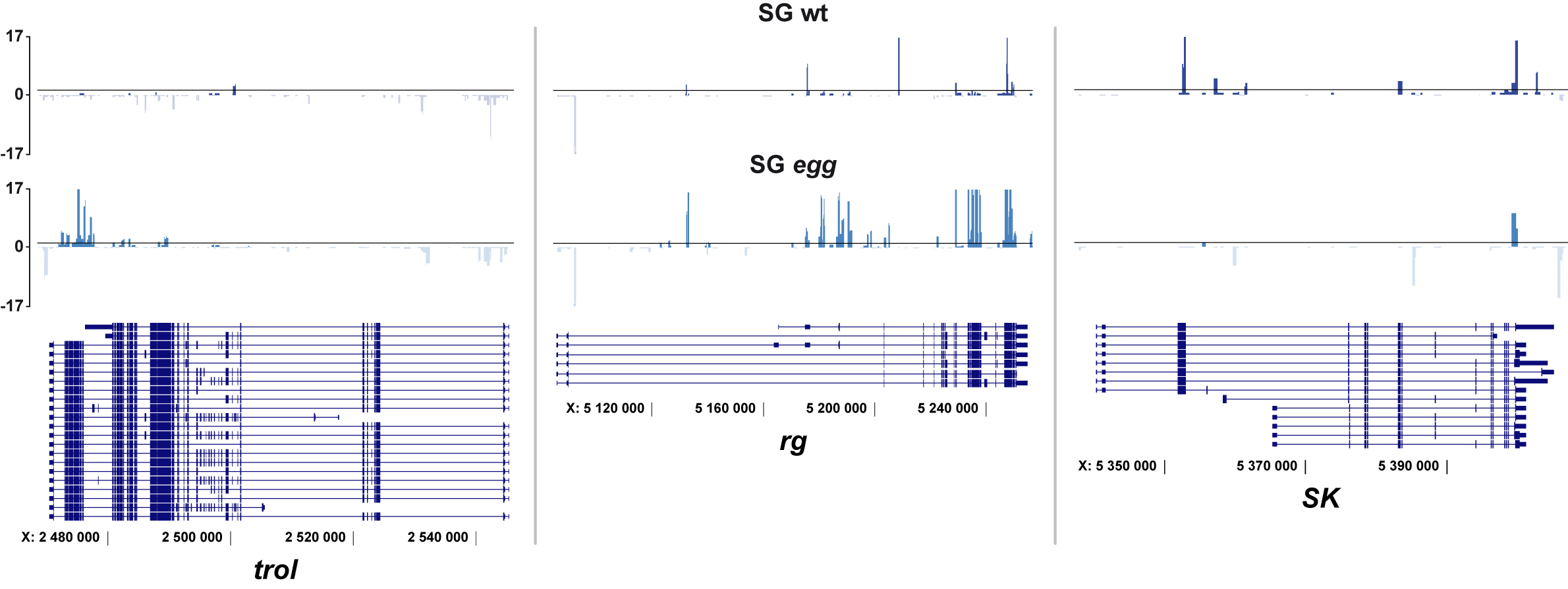

Supplement: Supplementary file 1 [file cells-08-01030-s001.zip › cells-562780-supplementary/cells-562780_Fig_S3.jpg]

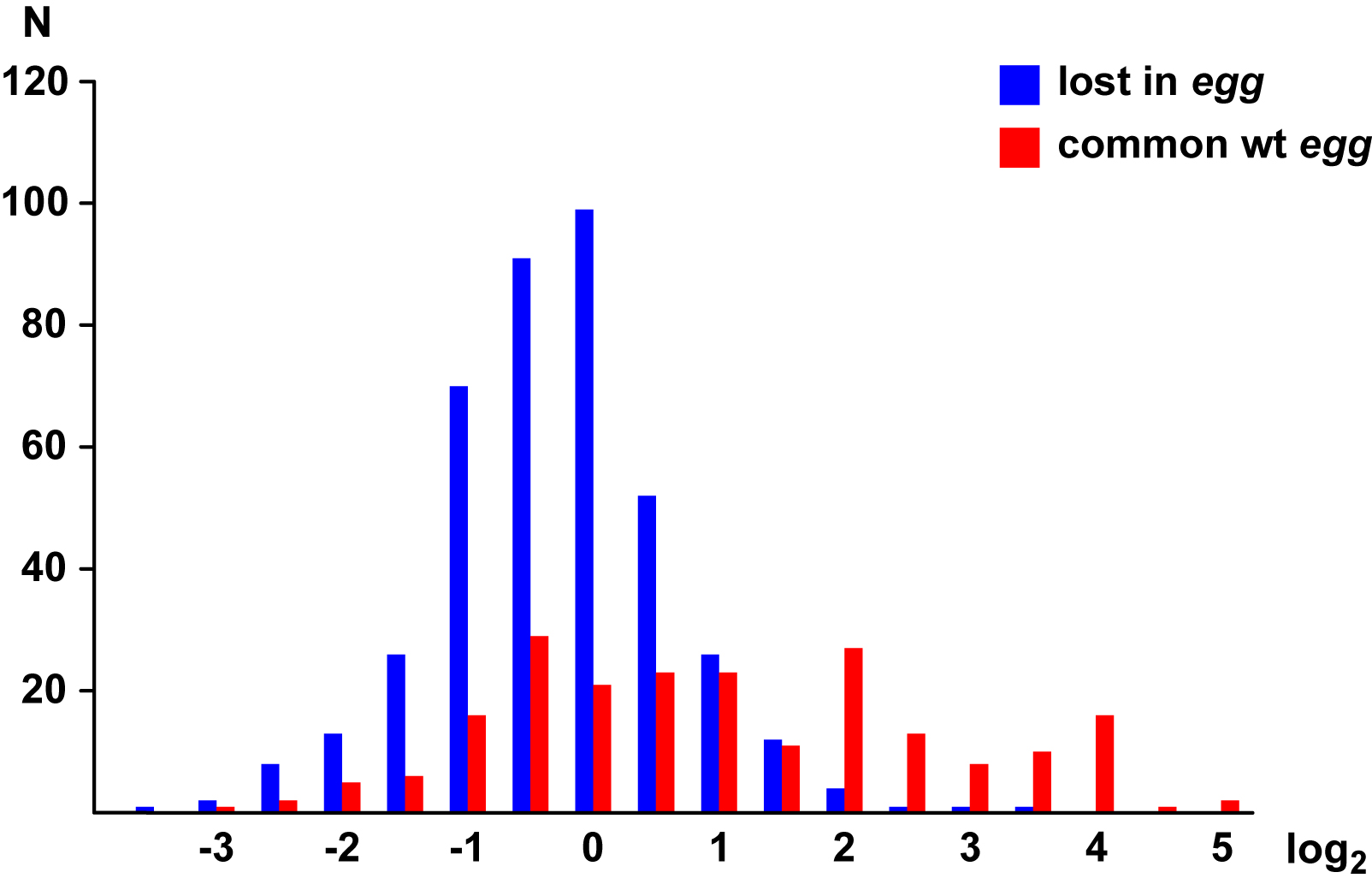

Supplement: Supplementary file 1 [file cells-08-01030-s001.zip › cells-562780-supplementary/cells-562780_Fig_S4.jpg]
